# Effects of soil nitrogen (N) deficiency on photosynthetic N-use efficiency in N-fixing and non-N-fixing tree seedlings in subtropical China

**DOI:** 10.1038/s41598-019-41035-1

**Published:** 2019-03-14

**Authors:** Jingchao Tang, Baodi Sun, Ruimei Cheng, Zuomin Shi, Da Luo, Shirong Liu, Mauro Centritto

**Affiliations:** 1 Key Laboratory on Forest Ecology and Environmental Sciences of State Forestry Administration, Institute of Forest Ecology, Environment and Protection, Chinese Academy of Forestry, Beijing, 100091 China; 2 School of Environmental and Municipal Engineering, Qingdao Technological University, Qingdao, 266033 China; 3 Co-Innovation Center for Sustainable Forestry in Southern China, Nanjing Forestry University, Nanjing, 210037 China; 4 Tree and Timber Institute, National Research Council of Italy, Via Madonna del Piano 10, 50019 Sesto Fiorentino (FI), Italy; 5 Research Institute of Economic Forestry, Xinjiang Academy of Forestry Science, Urumqi, 830000 China

## Abstract

Soil nitrogen (N) deficiencies can affect the photosynthetic N-use efficiency (PNUE), mesophyll conductance (*g*_m_), and leaf N allocation. However, lack of information about how these physiological characteristics in N-fixing trees could be affected by soil N deficiency and the difference between N-fixing and non-N-fixing trees. In this study, we chose seedlings of two N-fixing (*Dalbergia odorifera* and *Erythrophleum fordii*) and two non-N-fixing trees (*Castanopsis hystrix* and *Betula alnoides*) as study objects, and we conducted a pot experiment with three levels of soil N treatments (high nitrogen, set as Control; medium nitrogen, MN; and low nitrogen, LN). Our results showed that soil N deficiency significantly decreased the leaf N concentration and photosynthesis ability of the two non-N-fixing trees, but it had less influence on two N-fixing trees. The LN treatment had lower *g*_m_ in *D. odorifera* and lower leaf N allocated to Rubisco (*P*_R_), leaf N allocated to bioenergetics (*P*_B_), and *g*_m_ in *B. alnoides*, eventually resulting in low PNUE values. Our findings suggested that the *D. odorifera* and *E. fordii* seedlings could grow well in N-deficient soil, and adding N may increase the growth rates of *B. alnoides* and *C. hystrix* seedlings and promote the growth of artificial forests.

## Introduction

Nitrogen (N) is one of the most important biological elements for plants because it is a component of amino acids, proteins, genetic materials, pigments, and other key organic molecules^1–3^. A shortage of N results in a marked decrease in plant photosynthesis in many crops, and the leaf N content has a good correlation with the photosynthetic capacity^4^ because up to 75% of leaf N is present in the chloroplasts, with most of it in the photosynthetic apparatus^5^. The photosynthetic N-use efficiency (PNUE, the ratio of the photosynthetic capacity to the leaf N) is frequently used as an important leaf trait for characterizing leaf photosynthetic economics, physiology and strategy^6^. Many researchers have attempted to improve our understanding of the inherent variation in PNUE under soil N deficiency^1,7,8^.

Mesophyll conductance to CO_2_ and N allocation in the photosynthetic apparatus of a leaf cell are important factors that explain the differences in the PNUE^9,10^. Mesophyll conductance affects the CO_2_ contents of the carboxylation site, thus influencing the photosynthetic capacity and PNUE^11,12^. The N used in the photosynthetic apparatus could be divided into three parts, namely Rubisco (ribulose-1,5-bisphosphate carboxylase/oxygenase), bioenergetics, and light-harvesting components^13^. Rubisco is involved in carbon reduction reactions, and it is the most abundant enzyme in photosynthesis^14,15^. N is invested in bioenergetics, limiting the capacity for electron transport and photophosphorylation, and N is also invested in the contents of chlorophyll a/b protein complexes associated with photosystems I (PSI) and II (PSII), influencing light harvesting^13^.

Furthermore, N is involved in other components of the leaf cell apart from the photosynthetic apparatus. Cell walls play an important role in the mechanical toughness of plant tissues^16^ and they accumulate a significant amount of N compounds, at up to 10% of cell wall materials^17,18^. Trade-offs might occur for N allocation to cell walls versus Rubisco^16,18^. However, some researchers have suggested that these trade-offs might only be intraspecific^19^ and present in species lacking leaf N^20,21^. N is also involved in carbonic anhydrases and aquaporins^22^, with carbonic anhydrases accounting for 0.5–2% of the total soluble leaf protein^23^. These proteins play a role in mesophyll conductance (*g*_m_) by changing the nature of the diffusing molecule^24^ and facilitating CO_2_ diffusion through membranes^25^. Cell walls could account for >50% of the total resistance and a variable proportion of CO_2_ diffusion in the mesophyll, significantly affecting the variation of the *g*_m_^26^.

Soil N deficiency could affect the leaf N content, photosynthesis, PNUE, *g*_m_, and leaf N allocation in many species. Many researchers have found that the *A*_max_′ (light-saturated net CO_2_ assimilation rate) and *N*_area_ (leaf N concentration per area) were decreased in N-deficient soil^1,11,12,27^. However, the changes in the PNUEs of different species under soil N deficiency were uncertain; the PNUE values increased^1,28^, decreased^27,29^, or showed no marked change^7^ along the N addition gradients. The *g*_m_ was also usually decreased with soil N deficiency^11,12,30^. A lower soil N content could result in smaller chloroplasts^31^, leading to a decreased chloroplast surface area facing the intercellular air spaces^32^ and an increased distance between the intercellular space and the catalytic site of Rubisco^12^. Adding N to the soil could improve the leaf N content in the Rubisco, bioenergetics, and light-harvesting components^7,33–35^, but the changes in the proportion of N in these components were unclear^1,11^.

*Dalbergia odorifera*, *Erythrophleum fordii*, *Betula alnoides*, and *Castanopsis hystrix* are suitable species for forestation in southern subtropical China, and they have high economic value^36–39^. *D. odorifera* and *E. fordii* are N-fixing trees and *B. alnoides* and *C. hystrix* are non-N-fixing trees. Recent studies have found that Leguminosae trees with a higher *N*_area_ did not have a higher *A*_max_′ than other non-N-fixing species^40,41^. One possible explanation was that the Leguminosae tree species might allocate less N to Rubisco and bioenergetics than nonlegumes, as shown in previous studies^40–42^. However, there is a lack of information on how the leaf N content, leaf N allocation, mesophyll conductance to CO_2_ and PNUE of N-fixing trees could be affected by a low soil N content^43^.

In this study, we investigated the PNUE, photosynthesis, leaf N allocation and mesophyll conductance to CO_2_ in *D. odorifera*, *E. fordii*, *B. alnoides* and *C. hystrix* seedling leaves that were exposed to different soil N treatments. The objectives of our study were to 1. understand the effects of soil N deficiency on the PNUE, photosynthesis, leaf N allocation, and *g*_m_ of these trees; and 2. explore the different plant metabolism response modes between N-fixing and non-N-fixing woody species under soil N deficiency. We assumed that the photosynthetic capacity, PNUE and *g*_m_ of these trees might be reduced under a soil N deficiency, but the N-fixing trees were less affected.

## Results

### Effects of soil N treatments on *A*_max_′, *N*_area_, leaf N content per mass (*N*_mass_), leaf mass per area (LMA), and PNUE

**Figure 1:**
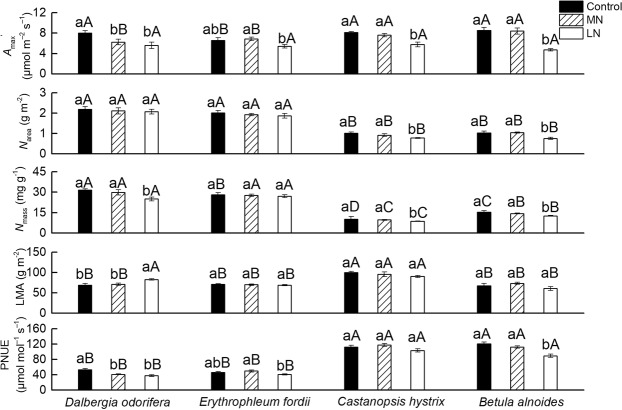
Light-saturated photosynthesis (*A*_max_′), leaf N content per area (*N*_area_), leaf N content per mass (*N*_mass_), leaf mass per area (LMA), and photosynthetic-N use efficiency (PNUE) in the seedling leaves from the four studied tree species after exposure to different soil nitrogen (N) treatments. The statistical differences between each characteristic of the different species under three N treatments (mean ± SE) are the results of a one-way analysis of variance (ANOVA) (n = 7). The lowercase letters indicate significant differences at the 0.05 level between different N treatments, and the uppercase letters indicate significant differences at the 0.05 level between the species under the same N treatment. Control, high N; MN, medium N; and LN, low N.

**Figure 2:**
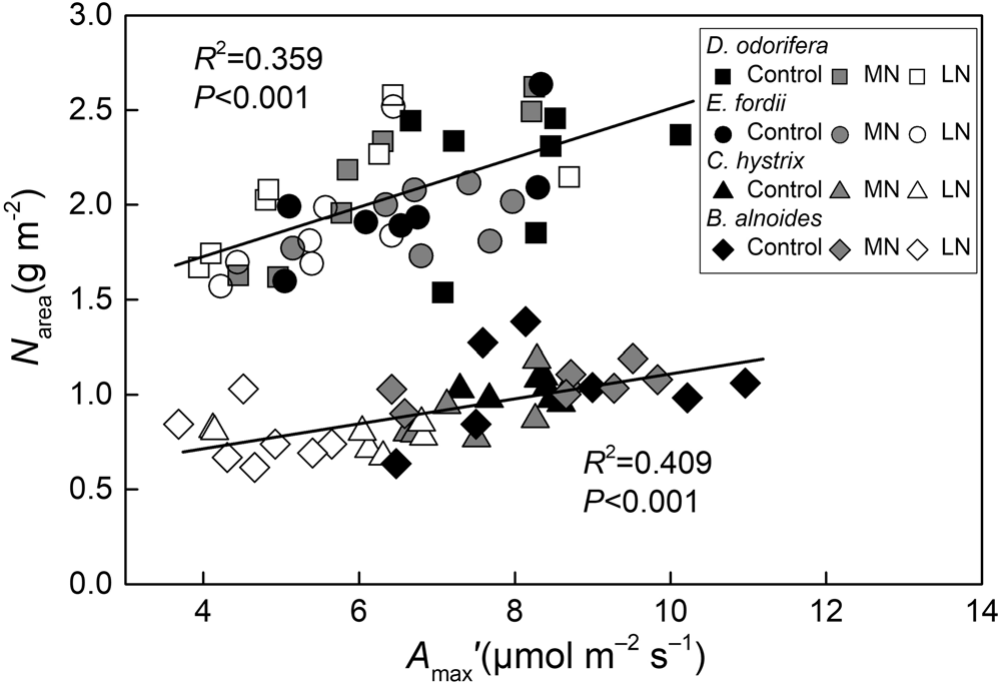
Regression analysis of the leaf nitrogen (N) concentration per area (*N*_area_) and light-saturated photosynthesis (*A*_max_′) of the seedling leaves from the four studied tree species. The determination coefficients (*R*^2^) and *P*-values are shown. The lines fitted for N-fixing and non-N-fixing trees are significantly different (*P* < 0.05) according to the result of a one-way analysis of covariance (ANCOVA) with *A*_max_′ as a dependent variable, whether it could fix N as a fixed factor, and *N*_area_ as a covariate

The seedling leaf *N*_area_ and *N*_mass_ values were significant higher in *D. odorifera* and *E. fordii* than they were in *C. hystrix* and *B. alnoides* under all the soil N treatments, and the PNUE was significantly lower in *D. odorifera* and *E. fordii* than it was in *C. hystrix* and *B. alnoides* (Fig. 1). The higher *N*_area_ and *N*_mass_ were direct causes of the lower PNUE in the two N-fixing tree seedlings. A significant decrease was observed in the *A*_max_′, *N*_mass_, and PNUE in the *D. odorifera*, *C. hystrix*, and *B. alnoides* seedling leaves under the low N treatments when compared with the high N conditions, and a significant decrease was observed in the *N*_area_ in the *C. hystrix* and *B. alnoides* seedling leaves (Fig. 1). The *A*_max_′, *N*_mass_, *N*_area_, LMA and PNUE of *E. fordii* were less affected by the soil N deficiency (for more details, see Supplementary Table S1). The *A*_max_′ had a significantly positive correlation with the *N*_area_ in these tree seedling leaves (*P* < 0.001; Fig. 2), which showed the importance of N on photosynthesis.

### Effects of soil N treatments on stomatal conductance (*g*_s_), *g*_m_, CO_2_ concentration in substomatal cavities (*C*_i_), CO_2_ concentration at the carboxylation site (*C*_c_), and *C*_i_–*C*_c_

**Figure 3:**
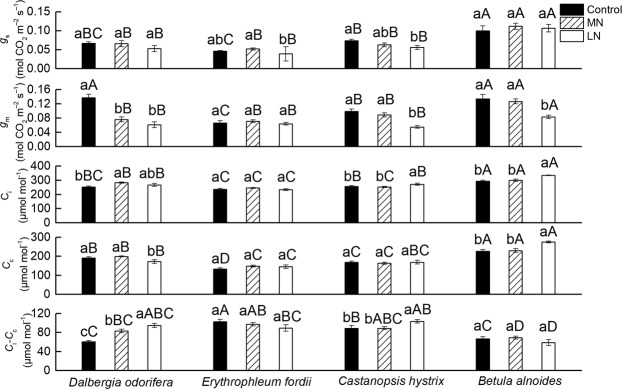
Stomatal conductance (*g*_s_), mesophyll conductance (*g*_m_), CO_2_ concentration in substomatal cavities (*C*_i_), CO_2_ concentration at the carboxylation site (*C*_c_), and *C*_i_–*C*_c_ in the seedling leaves of the four tree species after exposure to different soil nitrogen (N) treatments. The statistical differences between each characteristic of the different species under the three N treatments (mean ± SE) are the results of a one-way analysis of variance (ANOVA) (n = 7). The CO_2_ conductance data were measured under light saturated conditions, and the leaf chamber CO_2_ concentration was 380 μmol mol^−1^. The lowercase letters indicate significant differences at the 0.05 level between different N treatments, and the uppercase letters indicate significant differences at the 0.05 level between the species under the same N treatment. Control, high N; MN, medium N; and LN, low N.

The *g*_s_, *g*_m_, *C*_i_, and *C*_c_ in the *B. alnoides* seedling leaves were higher than they were in the other three species under any soil N treatments, except for the *g*_m_ under Control, and the *C*_i_–*C*_c_ of *B. alnoides* seedling leaves was lower than that of the other three species, except under Control (Fig. 3). This finding may be related to the fact that *B. alnoides* is a deciduous tree. The *g*_m_ and *C*_c_ of *D. odorifera* were significantly lower under LN than Control (−55.5% and −9.7%, respectively), but the *C*_i_–*C*_c_ was significantly higher in the LN treatment than under Control (+56.3%). No significant changes were observed in the *g*_s_, *g*_m_, *C*_i_, *C*_c_, and *C*_i_–*C*_c_ between Control and LN for *E. fordii*. The *g*_s_ and *g*_m_ of *C. hystrix* were significantly lower under LN than Control (−24.3% and −44.4%, respectively), but the *C*_i_ and *C*_i_–*C*_c_ were significantly higher under LN than Control (+5.6% and +14.8%, respectively). The *g*_m_ of *B. alnoides* was significantly lower under LN than Control (−38.0%), but the *C*_i_ and *C*_c_ were significantly higher under LN than Control (+14.2% and +21.7% Fig. 3). Different species have different response characteristics to the soil N conditions (More details see Supplementary Table S2).

### Effects of soil N treatments on maximum carboxylation rate (*V*_cmax_) and maximum electron transport rate (*J*_max_)

**Figure 4:**
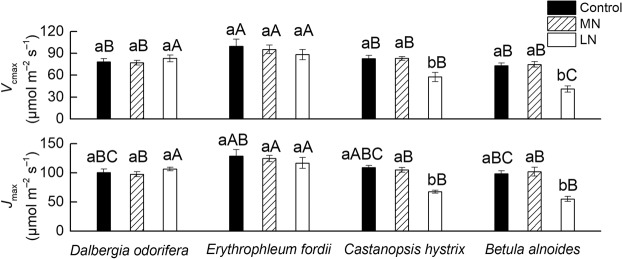
Maximum carboxylation rate (*V*_cmax_) and maximum electron transport rate (*J*_max_) in the seedling leaves of the four tree species after exposure to different soil nitrogen (N) treatments. The statistical differences between each characteristic of the different species under the three N treatments (mean ± SE) are the results of a one-way analysis of variance (ANOVA) (n = 7). The lowercase letters indicate significant differences at the 0.05 level between different N treatments, and the uppercase letters indicate significant differences at the 0.05 level between the species under the same N treatment. Control, high N; MN, medium N; and LN, low N.

The *V*_cmax_ values of *E. fordii* were significantly higher than those of the other three tree species under the Control and MN treatments. The *J*_max_ values of *E. fordii* were higher than those of the other three tree species only under MN treatment (Fig. 4). No significant difference was observed in the *V*_cmax_ and *J*_max_ of the *D. odorifera* and *E. fordii* seedling leaves between the different N treatments. The *V*_cmax_ and *J*_max_ of *C. hystrix* in the LN treatments were 30.5 and 38.1% significantly lower than those obtained from the Control treatment, and the *V*_cmax_ and *J*_max_ of *B. alnoides* were 43.7 and 43.7% significantly lower than those obtained under the Control treatment (Fig. 4). The *V*_cmax_ and *J*_max_ of the two N-fixing tree seedlings were less affected by the soil N deficiency (More details see Supplementary Table S3).

### Effects of soil N treatments on leaf N allocation proportion of the Rubisco (*P*_R_), bioenergetics (*P*_B_), light-harvesting components (*P*_L_), photosynthetic system (*P*_P_), cell wall (*P*_CW_), and other parts (*P*_Other_)

**Figure 5:**
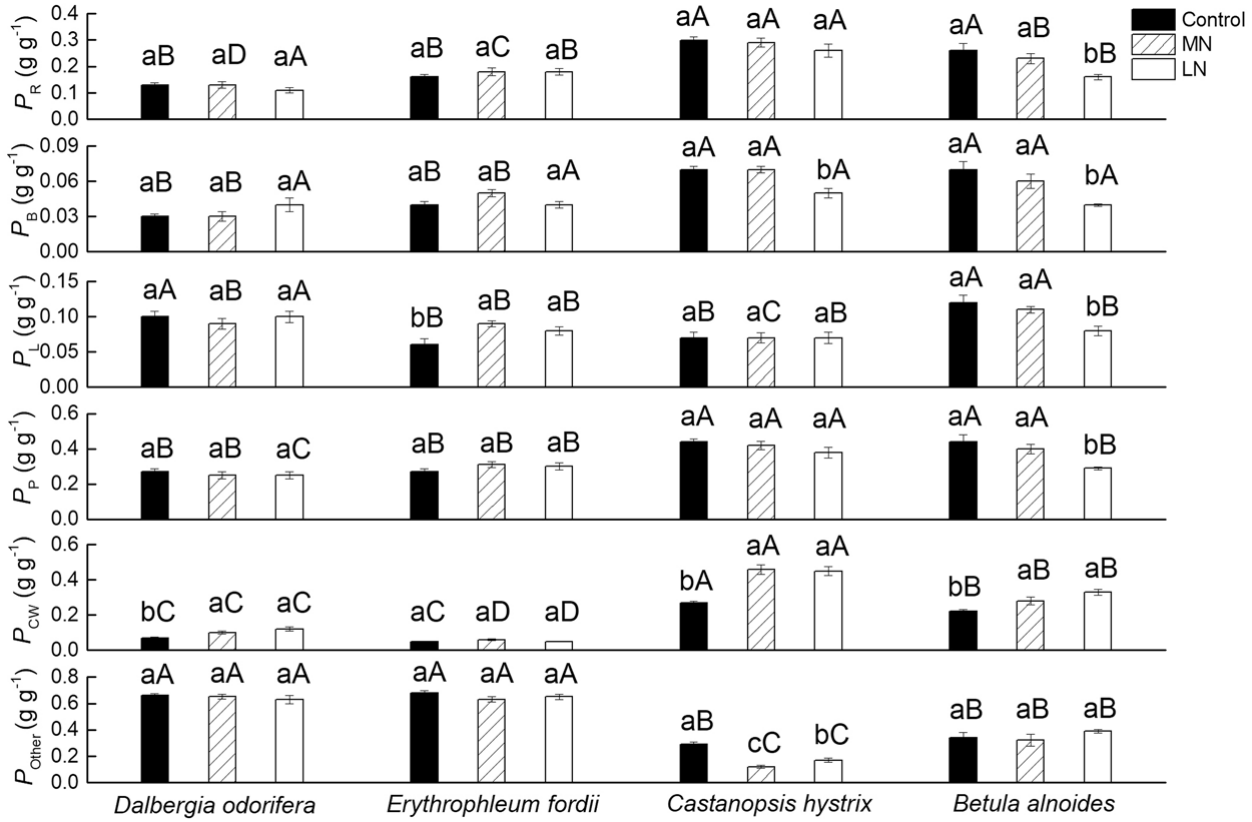
Nitrogen (N) allocation proportion of the Rubisco (*P*_R_), bioenergetics (*P*_B_), light-harvesting components (*P*_L_), photosynthetic system (*P*_P_), cell wall (*P*_CW_), and other parts (*P*_Other_) in the seedling leaves of the four tree species following exposure to different soil N treatments. The statistical differences between each characteristic of the different species under three N treatments (mean ± SE) are the results of a one-way analysis of variance (ANOVA) (n = 7). The lowercase letters indicate significant differences at the 0.05 level between different N treatments, and the uppercase letters indicate significant differences at the 0.05 level between the species under the same N treatment. Control, high N; MN, medium N; and LN, low N.

The *P*_R_, *P*_B_, *P*_P_ , and *P*_CW_ values of *C. hystrix* were higher than the corresponding values obtained for the other three species under any soil N treatments (Fig. 5). No significant change was observed in the *P*_R_, *P*_B_, *P*_L_, *P*_P_ , and *P*_Other_ values of *D. odorifera* under any N treatment; the *P*_CW_ of *D. odorifera* in the LN treatment was 71.4% higher than that in the Control treatment. No significant change was observed in the *P*_R_, *P*_B_, *P*_P_ , *P*_CW_, and *P*_Other_ values of *E. fordii* under any N treatments, and the *P*_L_ of *E. fordii* was 33.3% higher in the LN treatment than in the Control treatment. The LN treatment significantly decreased the *P*_B_ (−28.6%) and *P*_Other_ (−41.2%), and it increased the *P*_CW_ (+66.7%) of *C. hystrix* when compared with the corresponding values obtained under the Control conditions. The LN treatment significantly decreased the *P*_R_ (−38.5%), *P*_B_ (−42.9%), *P*_L_ (−33.3%), and *P*_P_ (−34.1%), and it increased the *P*_CW_ (+33.3%) of *B. alnoides* (Fig. 5). Overall, the N allocation of the two N-fixing tree seedlings changed little, but there was a large change for the two non-N-fixing tree seedlings (for more details, see Supplementary Table S4).

### Relationships between parameters

**Figure 6:**
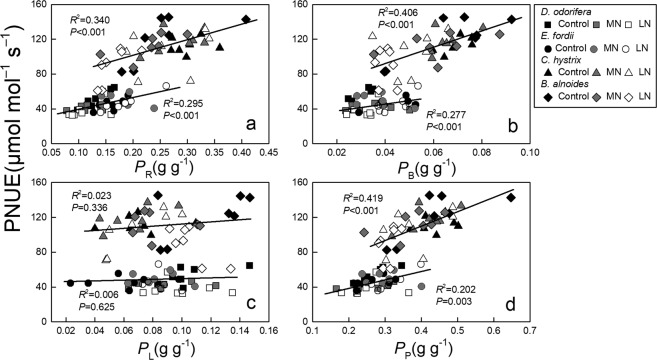
Regression analysis of nitrogen (N) allocation proportions in the photosynthetic system (*P*_P_), light-harvesting components (*P*_L_), Rubisco (*P*_R_), and bioenergetics (*P*_B_) with the photosynthetic N use efficiency (PNUE) in the seedling leaves of the four tree species after exposure to different soil N treatments. The determination coefficients (*R*^2^) and *P*-values are shown. The lines fitted for the N-fixing and non-N-fixing trees are significantly different (*P* < 0.05) according to the results of a one-way analysis of covariance (ANCOVA) with the PNUE as a dependent variable, whether it could fix nitrogen as a fixed factor, and *P*_P_, *P*_R_, *P*_B_, and *P*_L_ as covariates.

**Figure 7:**
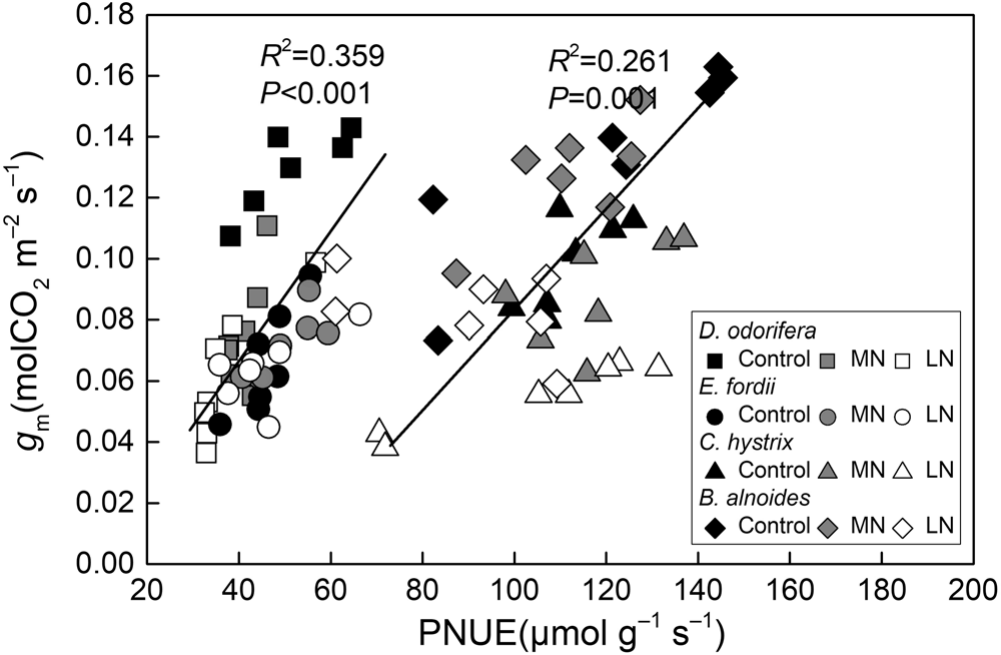
Regression analysis of *g*_m_ (mesophyll conductance) with the PNUE (photosynthetic nitrogen [N] use efficiency) in the seedling leaves of four tree species following exposure to different soil N treatments. The determination coefficients (*R*^2^) and *P*-values are shown. The lines fitted for the N-fixing and non-N-fixing trees are significantly different (*P* < 0.05) according to the results of a one-way analysis of covariance (ANCOVA) with the PNUE as a dependent variable, whether it could fix nitrogen as a fixed factor, and *g*_m_ as a covariate.

The *P*_R_, *P*_B_, and *P*_P_ values showed a significant positive correlation with the PNUE in these tree seedling leaves (*P* < 0.01; Fig. 6a,b,d). No significant correlation was observed between the *P*_L_ and PNUE in these trees (Fig. 6c). Significant positive relationships were observed between the *g*_m_ and PNUE in these tree seedling leaves (*P* ≤ 0.001; Fig. 7). The changes in *P*_R_, *P*_B_, and *g*_m_ were important physiological factors influencing the PNUE.

**Figure 8:**
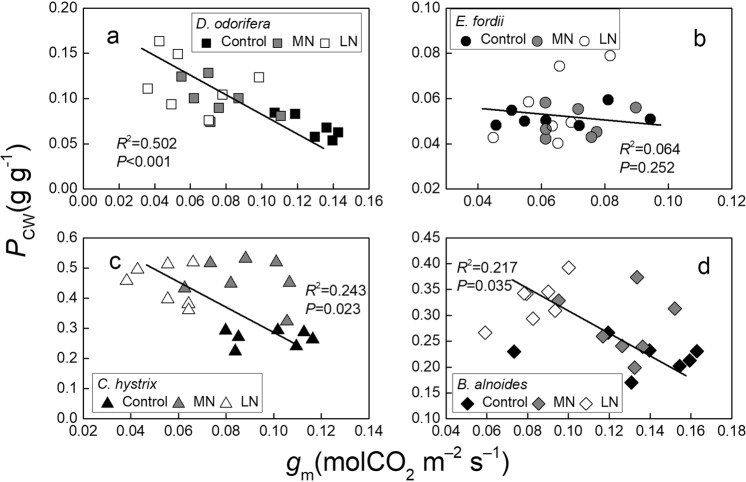
Regression analysis of the *g*_m_ (mesophyll conductance) with the *P*_CW_ (nitrogen [N] allocation proportion of cell wall) in the seedling leaves of four tree species under exposure to different soil N treatments. The determination coefficients (*R*^2^) and *P*-values are shown.

**Figure 9:**
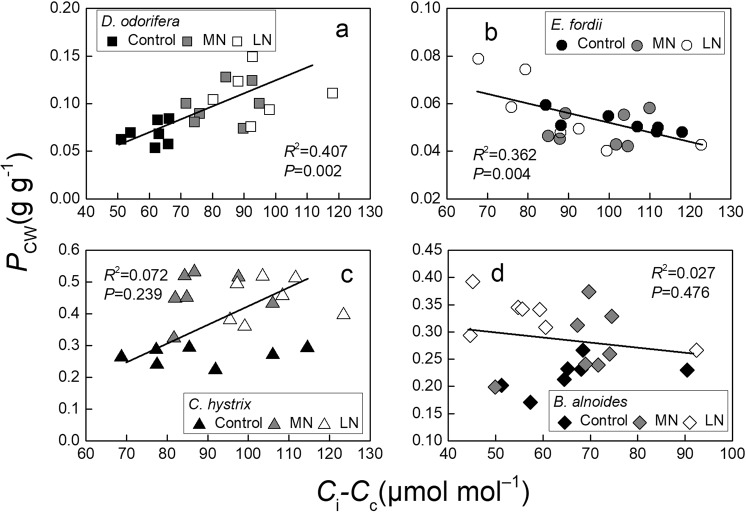
Regression analysis of *C*_i_–*C*_c_ (the difference between the CO_2_ concentration in the substomatal cavities [*C*_i_] and carboxylation site ([*C*_c_]) with the *P*_CW_ (nitrogen [N] allocation proportion of cell wall) in the seedling leaves of the four tree species under exposure to different soil N treatments. The determination coefficients (*R*^2^) and *P*-values are shown.

Significant negative relationships were found between the *P*_CW_ and *g*_m_ in *D. odorifera*, *E. fordii*, and *C. hystrix* (*P* < 0.001; Fig. 8a,c,d); no significant relationships were observed in *B. alnoides* (Fig. 8b). Significant positive relationships were observed between *P*_CW_ and *C*_i_–*C*_c_ in *D. odorifera* (*P* = 0.002; Fig. 9a). Significant negative relationships were noted between the *P*_CW_ and *C*_i_–*C*_c_ in *E. fordii* (*P* = 0.004; Fig. 9b), and no significant relationships were observed in *C. hystrix* and *B. alnoides* (Fig. 9c,d). The improved *P*_CW_ in *D. odorifera* might relate to its thicker cell walls, but in *E. fordii*, it might relate to the higher cell wall density.

**Figure 10:**
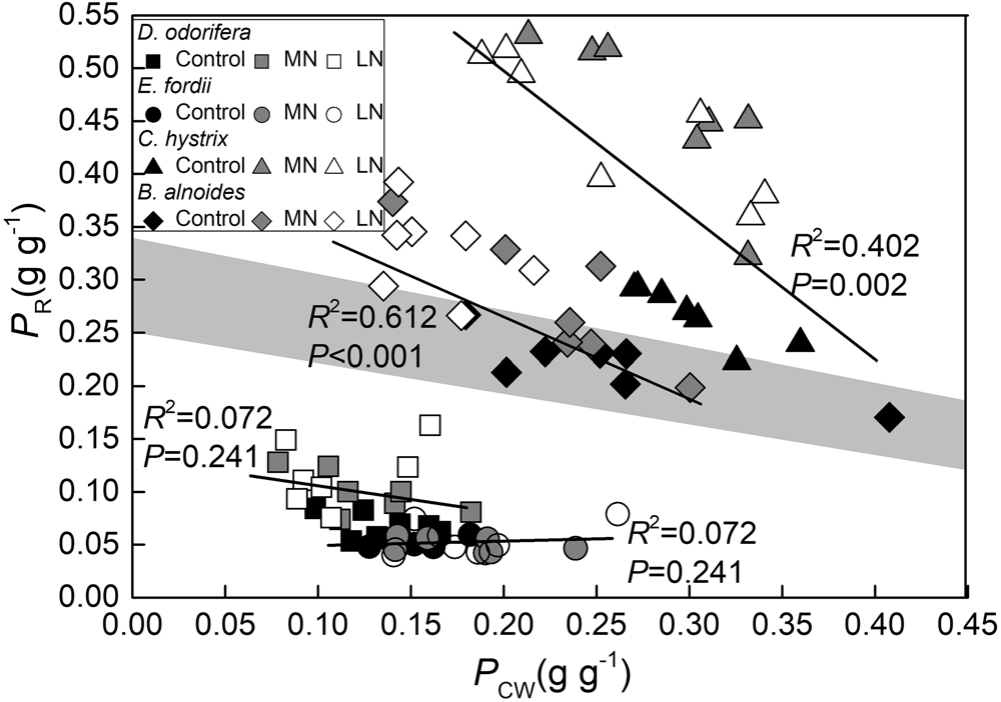
Regression analysis of the *P*_R_ (nitrogen [N] allocation proportion of Rubisco) and *P*_CW_ (N allocation proportion of cell wall) in the seedling leaves of the four tree species after exposure to different soil N treatments. The determination coefficients (*R*^2^) and *P*-values are shown. The shaded zone represents the distribution area of the *P*_CW_ and *P*_R_ in the presence of the trade-off^20^.

No significant relationships were observed between the *P*_CW_ and *P*_R_ in *D. odorifera* and *E. fordii*, but significant negative relationships were observed in *B. alnoides* and *C. hystrix* (*P* ≤ 0.002). The cell wall N might influence the variation in N in the Rubisco, thus influencing the photosynthetic capacity in these two non-N-fixing tree seedlings. A regression analysis of the *P*_CW_ with *P*_R_ in the *B. alnoides* seedling leaves under the LN treatment was obtained within the shaded zone. Most Control and MN treatment parameters for *B. alnoides* and *C. hystrix* were in the shaded zone, and *D. odorifera* and *E. fordii* were found under the shaded zone (Fig. 10). Low soil N increased the competition between the Rubisco and cell wall N.

## Discussion

The leaf N contents of two non-N-fixing tree seedlings, *B. alnoides* and *C. hystrix*, were significantly affected by the soil N content (Fig. 1, Supplementary Table S5), which was consistent with previously published studies^1,11,12,27^. However, the leaf N content of *E. fordii* was not significantly affected by the soil N content. This finding might be due to its strong N fixation capacity and its maintenance of the N content stability in leaves. Different N treatments significantly affected the *N*_mass_ of *D. odorifera* seedling leaves, but the *N*_area_ of *D. odorifera* was not affected by the soil N content (Fig. 1). Because the *N*_area_ was influenced by the *N*_mass_ and LMA, the LMA of *D. odorifera* changed with the soil N gradient (Fig. 1); the maintenance of the *N*_area_ at a steady state showed good leaf morphological plasticity. The low soil N content decreased the *A*_max_′ in *D. odorifera*, *B. alnoides*, and *C. hystrix* (Fig. 1) for different reasons. In *D. odorifera*, the low soil N content primarily decreased its *C*_c_ (Fig. 3), which is one of the important raw materials for photosynthesis^44^, and the CO_2_ partial pressure is important for Rubisco activity because O_2_ is a competitive inhibitor of the C assimilatory reaction of Rubisco for promoting the Rubisco oxidation reaction^12^. For the two non-N-fixing tree seedlings, the low soil N content decreased their *V*_cmax_ and *J*_max_ values (Fig. 4), which are the key biochemical parameters of the photosynthetic capacity^14,45^.

The fraction of the total leaf N allocated to the photosynthetic apparatus^46^, especially to Rubisco and bioenergetics, could influence the variation in the PNUE^1,3,16^. The *g*_m_ could also influence the PNUE^32,47^ by affecting the *C*_c_^11,12^. In this study, the *P*_R_ and *P*_B_ showed a significant positive correlation with the PNUE (*P* < 0.001, Fig. 6a,b), and the *g*_m_ significantly affected the PNUE in the seedling leaves of the four studied tree species (Fig. 7), although the effect of the *g*_m_ on the PNUE was different among the species^48^. The LN treatment significantly decreased the *g*_m_ in *D. odorifera* and the *P*_R_, *P*_B_, and *g*_m_ in *B. alnoides* (Figs 1 and 5), leading to lower PNUEs in the LN treatment. It has been reported that low soil N could decrease the *g*_m_^12,49^ and N allocation^3,29^. However, Chen *et al*. (2014) found an improvement in the *P*_R_ and *P*_B_ of female *Populus cathayana* with improved soil N, but the *P*_R_ and *P*_B_ of the males decreased^1^. Warren (2004) also found that an improvement in the soil N could decrease the *P*_R_ in *Eucalyptus globulus*. Some plants might have a different strategy for adapting to the soil N^11^.

The PNUEs of the two non-N-fixing tree seedlings were significantly higher than those of the two N-fixing tree seedlings under any soil treatment (Fig. 1, Supplementary Table S5), which was first attributed to their relatively low *N*_area_ and *N*_mass_ (Figs 1, 2, Table S7). The N-fixing species, which could gain N from air through legume bacteria, usually have a higher leaf N content than the non-N-fixing species^43,50^. High *P*_R_ and *P*_B_ (Fig. 4, Supplementary Table S5) were the primary biochemical factors leading to their higher PNUEs. These results were also consistent with other studies^40–42^. The leaves are the photosynthetic organs of plants, and plants have roughly two survival strategies, namely, quick investment-return and slow investment-return^51^. Two N-fixing trees might belong to the slow investment-return species and use a different strategy to use N, such as compensation for their low productivity through a long leaf lifespan^18^ and storing N for other processes, such as reproduction^1^. Two N-fixing tree seedlings might grow well in N-deficient soil, and applying N could increase the growth rates of the two non-N-fixing tree seedlings and promote the growth of artificial forests. Of course, some N-fixing trees have the same N utilization and distribution strategies as non-N-fixing trees, such as *Acacia mangium*^52^.

A decrease was observed in the *g*_m_ of the *D. odorifera*, *C. hystrix*, and *B. alnoides* seedlings under the LN treatment, but the reasons for this decline were different. The changes in *A*_max_′ or *C*_i_–*C*_c_ could influence the value of *g*_m_. In these tree seedlings, the *A*_max_′ decreased under the LN treatment, but the changes in the *C*_i_–*C*_c_ were different. *D. odorifera* and *C. hystrix* showed an increased *C*_i_–*C*_c_ in the LN treatment, but *B. alnoides* showed no change in its *C*_i_–*C*_c_ value (Fig. 3). After entering through the stomata, the CO_2_ diffuses through air spaces, cell walls, cytosol, and chloroplast envelopes and finally reaches the chloroplast stroma, where it is fixed by Rubisco^26,53^. Generally, cell walls account for >50% of the total cell CO_2_ diffusion resistance and a variable proportion of respiration^26^. *D. odorifera*, *C. hystrix*, and *B. alnoides* showed improved *P*_CW_ values in the LN treatment (Fig. 5). Mu *et al*. (2016) also found an increase in the *P*_CW_ of maize growing under low-N stress^29^. *D. odorifera* showed no significant reduction in its *N*_area_ in the LN treatment, and thus there was an increase in the N contents in the cell wall (*Q*_CWarea_) of *D. odorifera* (+62.4%, Supplementary Table S6). The percentage of N in the cell wall showed a slight variation in the same species^16^. An improvement in the *N*_CW_ of *D. odorifera* under the LN treatment indicates the high dry mass of the cell wall, resulting in improved LMA^16,54^, and it might improve the thickness of the cell wall, thereby improving its *C*_i_–*C*_c_ value^16^. However, *B. alnoides* and *C. hystrix* showed a reduction in their *N*_area_ values in the LN treatment, leading to a smaller change in the *Q*_CWarea_ (+5.9% and +29.6%, respectively, Supplementary Table S6). Thus, there were no significant changes in their LMA and *C*_i_–*C*_c_ values. An improvement in the *P*_CW_ of *D. odorifera* therefore significantly decreased its *C*_i_–*C*_c_ and *g*_m_, and no significant relationship was observed between the *P*_CW_ and *C*_i_–*C*_c_ in *B. alnoides* and *C. hystrix* (Figs 8, 9).

The *P*_CW_ did not influence the variation in the *C*_i_–*C*_c_, but it showed a significant negative correlation with the *g*_m_ in two non-N-fixing trees (Fig. 8). The cell wall N might influence the N variation in Rubisco, thus influencing the *V*_cmax_ and *A*_max_′ values. Onoda *et al*. (2004) and Takashima *et al*. (2004) observed a trade-off between the cell wall and Rubisco N in *Polygonum cuspidatum* and in *Quercus* species, respectively^16,18^. Zhang *et al*. (2016) also found this trade-off in *Mikania micrantha* and *Chromolaena odorata*^28^. Hikosaka and Shigeno (2009) considered this relationship unlikely to hold as a general rule; the allocation of N to the cell walls did not explain the variation in the Rubisco^19^. Harrison *et al*. (2009) and Qing *et al*. (2012) believed that this relationship might occur during N leaf deficiency^20,21^. *B. alnoides* and *C. hystrix* showed high *P*_R_ and *P*_CW_ values (Fig. 5), and a part of the distribution area in or on the shade zone (Fig. 10; for a further explanation of the shade zone, please see Harrison *et al*.^20^) indicates that the free amino acid, NO_3_^−^, and NH_4_^+^ in the leaves were not sufficient (appearing as low *P*_Other_) to supply N to both Rubisco and the cell wall^20^, which explained the existence of a trade-off between the *P*_R_ and *P*_CW_ (Fig. 10). It is important to note that the regression analysis of the *P*_CW_ with the *P*_R_ in the *B. alnoides* seedling leaves exposed to the LN treatment was found in the shaded zone; most Control and MN treatments of *B. alnoides* and *C. hystrix* were in the shaded zone (Fig. 10). Low soil N increased the competition between the Rubisco and cell wall N.

The two non-N-fixing tree seedling leaves showed improved *P*_CW_, and the *D. odorifera* seedling leaves improved both the LMA and *P*_CW_ values under the LN treatment (Figs 1, 5). The LMA is the product of leaf thickness and density, and it is positively correlated with leaf toughness^55^ and is a fundamental defensive trait of plants^56,57^. The cell wall also directly functions as a defense organ^58^. We observed that the *N*_mass_ values of these trees were affected by the soil N content (Fig. 1). Low nutrient availability limits the growth rate of seedlings and might damage the seedlings during the growing season^59^. The LN treatment might pose a threat to these seedling leaves; thus, plants need to have tougher leaves to survive^16^, as shown by the relatively high *P*_CW_ and LMA in *D. odorifera* seedling leaves and high *P*_CW_ in the *B. alnoides* and *C. hystrix* seedling leaves. Givnish (2002) hypothesized that soil fertility is the primary driver of the leaf lifespan^60^, and a high LMA leads to a long leaf lifespan^51^. Therefore, an improvement in the LMA might also increase the leaf lifespan of *D. odorifera* seedling leaves, ultimately maximizing the carbon assimilation per unit of nutrient over the lifespan of the leaf^61–63^. Different species have different response characteristics to the soil N conditions.

**Figure 11:**
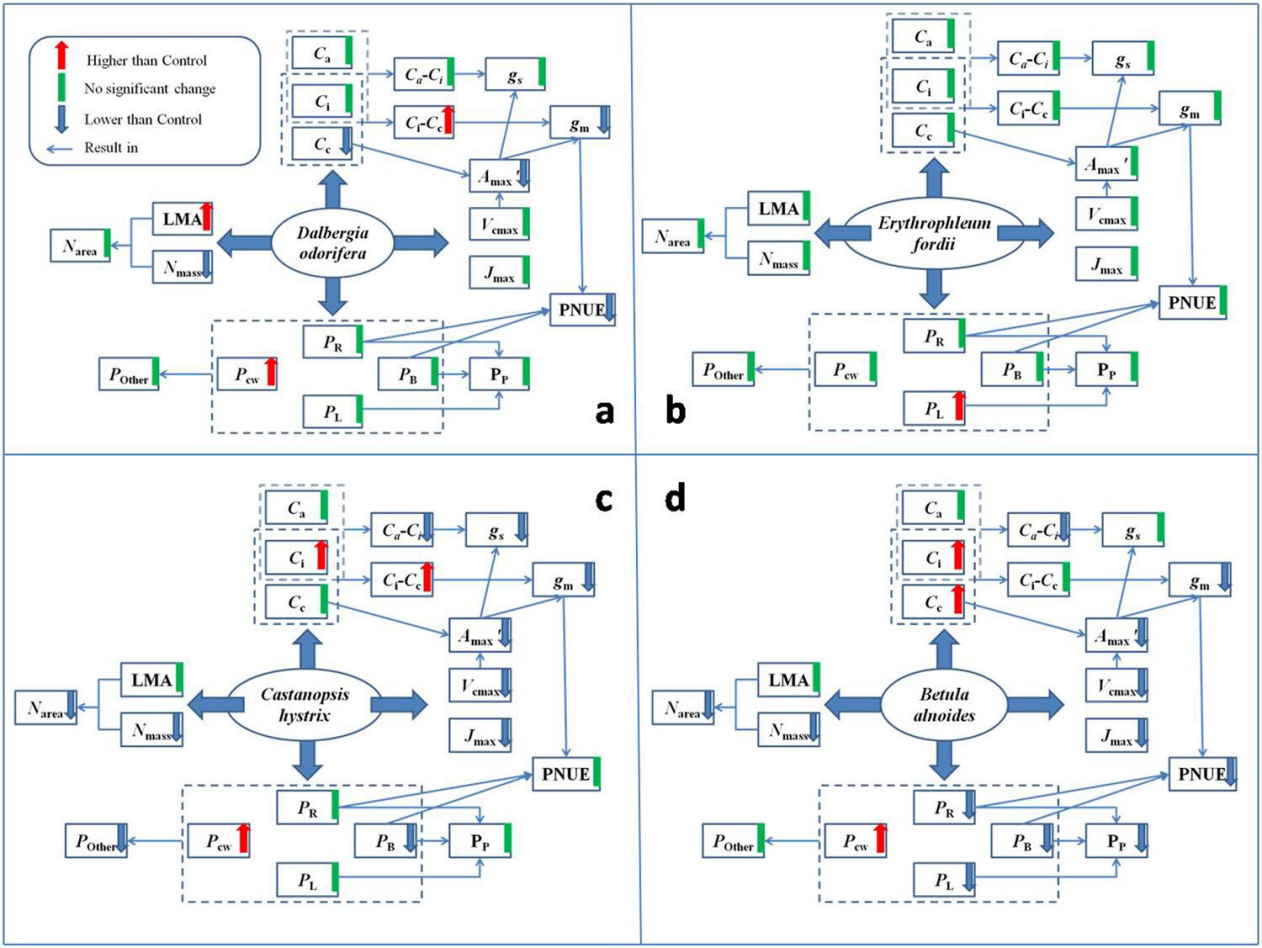
Changes in the variables under low soil nitrogen in four species.

To understand the changes in the various parameters under low soil N in the four species, we drew a process diagram (Fig. 11). Generally, we found fewer parameter changes in the two N-fixing tree seedlings and more parameter changes in the two non-N-fixing tree seedlings. The physiological and ecological characteristics of these two N-fixing tree seedlings are more stable, and these two N-fixing tree seedlings could be good tree species for afforestation in N-poor areas. We also performed Between-Subjects effects tests on the tree varieties and N treatments for the variables in the four species (Supplementary Table S8). In general, varieties of the trees were more important than the N treatment interaction effect, but the N treatment interaction effect was more important in influencing the *A*_max_′ and *g*_m_. More trees and more variables must be further studied.

## Conclusions

In revisiting our questions, we concluded that (1) soil N deficiency significantly decreased the leaf N concentration and photosynthesis ability in two non-N-fixing trees, but it had less influence on these indices in the two N-fixing trees. (2) The LN treatment had a lower *g*_m_ in *D. odorifera* and had lower *P*_R_, *P*_B_ and *g*_m_ in *B. alnoides*, eventually resulting in their low PNUE values. (3) *D. odorifera*, *B. alnoides*, and *C. hystrix* seedling leaves showed improved *P*_CW_ and (or) LMA to adapt to a low-N soil environment. These findings were important for understanding the ecophysiological changes in plants under low soil N conditions. Our findings suggested that the two N-fixing tree seedlings could grow well in N-deficient soil, and they could be good tree species for the afforestation of N-poor areas. Adding N may increase the growth rates for the two non-N-fixing tree seedlings and promote the growth of artificial forests. Because these species live in the same area, it is possible to mix non-N-fixing with N-fixing tree seedlings for afforestation, and mix N-fixing trees in non-N-fixing pure forest after intermediate cutting or selective cutting in non-N-fixing pure forest, which could improve soil N utilization efficiency.

## Materials and Methods

### Study area and plant material

This study was performed in the Experimental Center of Tropical Forestry (22°7′19″–22°7′22″N, 106°44′40″–106°44′44″E) at the Chinese Academy of Forestry located in Pingxiang, Guangxi Province, China. This location has a subtropical monsoon climate with distinct dry and wet periods, and the mean annual temperature is 21 °C. The mean monthly minimum and maximum temperatures are 12.1 and 26.3 °C, respectively. The mean annual precipitation, which takes place primarily from April to September, is 1400 mm. The active accumulated temperature above 10 °C is 6000–7600 °C. The total annual sunshine duration is 1419 hours^64,65^.

The seeds of *D. odorifera*, *E. fordii*, and *C. hystrix* were collected separately from the mother trees, and the *B. alnoides* seedlings were somaclones. The *D. odorifera*, *E. fordii*, and *C. hystrix* seeds were germinated in a seedbed in February of 2014, and *B. alnoides* was budding at the same time. When the seedlings were approximately 20 cm tall, 90 similarly sized seedlings per species were transplanted to pots (5.4 L, filled with washed river sand) and established in an open site at the Experimental Center of Tropical Forestry in March, 2014.

From April to June, three levels of soil N treatments were set up (Hyponex M. Scott & Sons, Marysville, OH, USA, dissolved in the water from the aqueous solution preparation). Nitrogen fertilizer was applied ten times, once per week. A total of 0.2 (low nitrogen, LN), 0.7 (medium nitrogen, MN), and 1.5 g (set as Control) of available N were applied per pot, with each treatment including 30 seedlings per species. The forms of N that were applied in this study were mixed N (both NH_4_^+^ and NO_3_^−^), and the NH_4_^+^ to NO_3_^−^ ratio was 1:1. We chose these forms because we used washed river sand as a culture substrate with a pH value of approximately 7, and only using NH_4_^+^ or NO_3_^−^ might cause the soil to become more acid or alkaline, respectively, affecting the plant growth. Wu *et al*. (2012) found that the proper amounts of N applications for *D. odorifera* seedlings were 1.74–2.15 g N per pot^66^. Li *et al*. (2003) found that the appropriate N applications for *E. fordii* seedlings were approximately 1.39–1.86 g N per pot^67^. Although the purpose of this research is to understand the effects of soil N deficiency on plant metabolism, we also want to explore the plant physiological process from a comparatively sufficient to a lack of soil N, because non-N-fixing woody species might be more sensitive to changes in the soil N gradient, and the different ecophysiological processes between a comparatively sufficient to a lack of soil N could help us to understand the effects of soil N deficiency on plant metabolism. Therefore, we set up a high N treatment as Control. The seedlings in each treatment were watered every day to keep the soil moist. Natural light (100% light in the field) was used for illumination.

### Determination of gas exchange parameters

Fifteen days after the last N fertilization, on sunny days from 9:00 to 11:00 h in July and August of 2014, seven healthy and similarly sized seedlings were chosen per treatment, per species. One healthy and mature leaf per seedling that was exposed to the sun was chosen to determine the gas exchange parameters. These parameters were determined with a LiCor-6400 portable photosynthesis system (LI-COR, Lincoln Nebraska, USA), and the photosynthetic response to the photosynthetic photon flux density (PPFD, µmol m^−2^ s^−1^) and *C*_i_ (μmol mol^−1^) were determined. Under 380 μmol mol^−1^ of leaf chamber CO_2_ concentration (the average air CO_2_ concentration in the day time), the photosynthetic rates were measured under photon flux densities of 1500, 1200, 1000, 800, 600, 400, 200, 150, 100, 80, 50, 30, 20, 10 and 0 μmol m^−2^ s^−1^. Under a saturated PPFD, the photosynthetic rates were detected using the same leaf-under leaf chamber CO_2_ concentrations of 380, 200, 150, 100, 80, 50, 380, 600, 800, 1000, 1200, 1500, 1800 and 2000 μmol mol^–1 ^^28,47^. We started at a 380 μmol mol^−1^ concentration because this is the average air CO_2_ concentration during the day time that could reduce the plant activation time^28^. The relative humidity of the air in the leaf chamber was maintained at 60–70%, and the leaf temperature was set to 30 °C. The values for the following data or parameters were determined: the net photosynthetic rate (*A*_n_, μmol m^−2^ s^−1^), *A*_max_′ (μmol m^−2^ s^−1^), *g*_s_ (mol CO_2_ m^−2^ s^−1^), and dark respiration (*R*_n_, μmol m^−2^ s^−1^). The light- and CO_2_-saturated net CO_2_ assimilation rate (*A*_max_, μmol m^−2^ s^−1^) was calculated according to Farquhar *et al*.^14^. The relative humidity of the air in the leaf chamber was maintained at 60–70%, and the leaf temperature was set to 30 °C.

### Determination of the chlorophyll fluorescence, mesophyll conductance, *V*_cmax_, and *J*_max_

The fluorescence yield was measured using a LiCor-6400 leaf chamber fluorometer (6400–40, LI-COR, Lincoln, Nebraska, USA) on the same leaf and with seven repetitions for each species. The chamber relative humidity and leaf temperature were controlled under the same conditions as described in the gas exchange parameters. The leaf chamber CO_2_ concentration was set to 380 μmol mol^−1^. The fluorescence yield (*ΔF*/*F*_m_′) was subsequently determined. The photosynthetic electron transport rate (*J*_f_, μmol m^−2^ s^−1^) was calculated according to the equation described by Loreto *et al*.^68^ as follows:

1 $${J}_{f}=PPFD\times \frac{{\rm{\Delta }}F}{{F}_{m}^{\prime} }\times Leafreflu\times PARDistPhotosys$$

where *PPFD* is the photosynthetic photon flux density; *Leafreflu* is the leaf absorptance valued between 0.82–0.85^69^ (we used 0.85 in this paper); and *PARDistPhotosys* is the fraction of quanta absorbed by photosystem II (valued as 0.5)^68^. The mesophyll conductance (*g*_m_, mol CO_2_ m^−2^ s^−1^) was calculated using three different methods to obtain a more accurate value. The variable *J* method was described by Harley *et al*.^70^, and it has been commonly used in recent years^71–73^. The *A*-*C*_i_ curve fitting method was described by Ethier and Livingston^74^, and Sharkey *et al*.^75^ developed a software package to estimate the *g*_m_ and other parameters based on this method. The exhaustive dual optimization (EDO) method described by Gu *et al*.^76^ could estimate up to eight parameters, including the *g*_m_, and we obtained an automated analysis of *A*-*C*_i_ curves through a website (http://www.leafweb.org) by uploading our data to determine the value of the *g*_m_. Subsequently, the *g*_m_ calculated by these three methods was used to calculate *C*_c_ (μmol mol^−1^) as follows:

2 $${C}_{C}={C}_{i}-\frac{{A}_{max}^{\prime} }{{g}_{m}}$$

The *C*_c_ and *g*_m_ calculated using the three methods are shown in Supplementary Table S9. The mean value of *C*_c_ was used to fit the *A*_*n*_-*C*_c_ curve, followed by the calculation of *V*_cmax_ (μmol m^−2^ s^−1^) according to Farquhar *et al*.^14^ and the *J*_max_ (μmol m^−2^ s^−1^) according to Loustau *et al*.^77^. The running fitting model used in the *in vivo* Rubisco kinetics parameters (i.e., *K*_o_, *K*_c_, and their activation energy) was measured according to Niinemets and Tenhunen^13^.

### Determination of additional leaf traits

After the gas exchange parameters and fluorescence yield were determined, the leaf samples and nearby leaves (30–50 leaves per seedling in total, the sizes of which were similar to those of the leaves used to determine the photosynthesis, healthy and mature characteristics, and sun-exposed parameters) were collected from each pot. The surface areas of 10–20 leaves were measured using a scanner (Perfection v700 Photo, Epson, Nagano-ken, Japan). The leaves were subsequently oven-dried to a constant weight at 80 °C for 48 h. The dry weight was measured using an analytic balance, and then the LMA (g m^−2^) was calculated. The dried leaf samples were ground into dry flour. The organic carbon (C) concentration was determined by potassium dichromate-sulfuric acid oxidation method (*C*_mass_ mg g^−1^, Supplementary Table S10). The N concentration was determined using a VELP automatic Kjeldahl N determination apparatus (UDK-139, Milano, Italy), and then the *N*_mass_ (mg g^−1^) and *N*_area_ (g m^−2^) values were calculated. Then, PNUE (μmol mol^−1^ s^−1^) was calculated using the following formula:

3 $${\rm{PNUE}}=\frac{{A}_{{\rm{\max }}}^{\prime} }{{N}_{{\rm{area}}}}\times 14$$

where 14 is the atomic mass of nitrogen.

The remaining 20–30 leaves were frozen and kept for laboratory analysis. The frozen leaves (0.2 g, 5–10 leaves) were cut into small 5–10-mg pieces. The leaves were placed in a volumetric flask and brought to a consistenttant volume of 25 mL using 95% (v/v) alcohol. The volumetric flask was protected from light for 24 h, and then the chlorophyll contents were determined using a Shimadzu ultraviolet-visible spectrophotometer (UV 2250, Fukuoka, Japan). For the chlorophyll contents, please see Supplementary Table S10.

The remaining frozen leaves were used to determine the cell wall N content according to the method of Onoda *et al*.^16^ as follows: 1 g of leaves was powdered in liquid N and suspended in sodium phosphate buffer (pH 7.5, 25 mL), the homogenate was centrifuged at 2500 g for 5 min, and the supernatant was discarded. The pellet was washed with 3% (w/v) SDS, amyloglucosidase (35 units ml^−1^, *Rhizopus* mold, Sigma, St Louis, USA) and 0.2 M KOH and then heated and centrifuged, and the remaining pellet was washed with distilled water and ethanol and then dried in an oven (75 °C) for 2 days (for more details see Onoda *et al*.)^16^. The nitrogen content of the rest of the pellet (cell wall N) was determined using a VELP automatic Kjeldahl N determination apparatus. The *P*_CW_ represents the ratio of the cell wall N content to the total N content.

### Calculation of the N allocation in the photosynthetic apparatus

The N allocation fractions of each component in the photosynthetic apparatus were calculated according to Niinemets and Tenhunen^13^, which has been widely used in recent years^1,45,78^.

4 $${P}_{{\rm{R}}}=\frac{{V}_{{\rm{cmax}}}}{6.25\times {V}_{{\rm{cr}}}\times {\rm{LMA}}\times {N}_{{\rm{mass}}}}$$

5 $${P}_{{\rm{B}}}=\frac{{J}_{{\rm{\max }}}}{8.06\times {J}_{{\rm{mc}}}\times {\rm{LMA}}\times {N}_{{\rm{mass}}}}$$

6 $${P}_{{\rm{L}}}=\frac{{C}_{{\rm{Chl}}}}{{C}_{{\rm{B}}}\times {N}_{{\rm{mass}}}}$$

where *C*_Chl_ is the chlorophyll concentration (mmol g^−1^), *V*_cr_ is the specific activity of Rubisco (μmol CO_2_ g^−1^ Rubisco s^−1^), *J*_mc_ is the potential rate of photosynthetic electron transport (μmol electrons μmol^−1^ Cyt f s^−1^), and *C*_B_ is the ratio of leaf chlorophyll to leaf N during light-harvesting (mmol Chl (g N)^−1^). The *V*_cr_, *J*_mc_, and *C*_B_ were calculated according to Niinemets and Tenhunen^13^.

### Statistical analysis

The differences between the seedling leaves of the four tree species, the N-fixing and non-N-fixing tree seedlings, and the three levels of soil N were analyzed by performing a one-way analysis of variance (ANOVA), and a post-hoc test (Tukey’s test) was conducted to determine if the differences were significant. The effects of the tree varieties and N treatments on the variables in the four species were analyzed by two-way ANOVA and Tukey’s test. The significance of the correlation between each pair of variables was tested with a Pearson’s correlation (two-tailed). All the analyses were performed using the Statistical Product and Service Solutions 17.0 program (SPSS17.0, Chicago, USA).

## Supplementary information

Table S1, Table S2, Table S3, Table S4, Table S5, Table S6, Table S7, Table S8, Table S9, Table S10

## Data Availability

All the relevant data are in the paper and its Supporting Information files.
