# Supplementary material for: Effects of soil nitrogen (N) deficiency on photosynthetic N-use efficiency in N-fixing and non-N-fixing tree seedlings in subtropical China
Source: Sci Rep. 2019 Mar 14;9:4604. doi: 10.1038/s41598-019-41035-1 (PMC6418086; doi:10.1038/s41598-019-41035-1)
Supplement: Supplementary file 1 — Table S1, Table S2, Table S3, Table S4, Table S5, Table S6, Table S7, Table S8, Table S9, Table S10 [file 41598_2019_41035_MOESM1_ESM.docx]

**Effects of** **soil nitrogen (N) deficiency on photosynthetic N-use efficiency in N-fixing and non-N-fixing tree seedlings in subtropical China**

**Jingchao Tang^1,2^, Baodi Sun^2^, Ruimei Cheng^1,3^, Zuomin Shi^1,3,4*^, Da Luo^1,5^, Shirong Liu^1^, &** **Mauro Centritto^4^**

**Table S1** Light-saturated photosynthesis (*A*_max_’), leaf N content per area (*N*_area_), leaf N content per mass (*N*_mass_), , leaf mass per area (LMA), and photosynthetic-N use efficiency (PNUE) in the seedling leaves of four studied tree species exposed to different soil nitrogen (N) treatments

| Tree species | N treatments | *A*_max_′ (μmol m^–2^ s^–1^) | *N*_area_ (g m^-2^) | *N*_mass_ (mg g^-1^) | LMA (g m^-2^) | PNUE (μmol mol^–1^ s^–1^) |
| --- | --- | --- | --- | --- | --- | --- |
| *Dalbergia odorifera* | Control | 8.04 ± 0.46^aA^ | 2.19 ± 0.13^aA^ | 31.70 ± 0.76^aA^ | 68.97 ± 3.90^bB^ | 52.64 ± 3.78^aB^ |
|  | MN | 6.26 ± 0.55^bB^ | 2.11 ± 0.15^aA^ | 29.88 ± 1.87^aA^ | 71.01 ± 3.14^bB^ | 41.02 ± 1.26^bB^ |
|  | LN | 5.58 ± 0.63^bA^ | 2.07 ± 0.11^aA^ | 25.04 ± 1.37^bA^ | 82.86 ± 1.73^aA^ | 37.38 ± 2.22^bB^ |
|  | *F* and *P* | *F* =5.332^*^, *P* =0.015 | *F* =0.178, *P* =0.837 | *F* =5.998^**^, *P* =0.011 | *F* =5.995^**^, *P* =0.010 | *F* =6.931^**^, *P* =0.006 |
| *Erythrophleum fordii* | Control | 6.60 ± 0.50^abB^ | 2.01 ± 0.12^aA^ | 28.09 ± 1.49^aB^ | 71.35 ± 0.89^aB^ | 45.92 ± 2.24^abB^ |
|  | MN | 6.86 ± 0.36^aB^ | 1.93 ± 0.06^aA^ | 27.62 ± 0.86^aA^ | 70.12 ± 1.88^aB^ | 49.84 ± 2.66^aB^ |
|  | LN | 5.41 ± 0.33^bA^ | 1.87 ± 0.12^aA^ | 27.06 ± 0.99^aA^ | 68.86 ± 1.77^aB^ | 40.60 ± 1.82^bB^ |
|  | *F* and *P* | *F* =3.645^*^, *P* =0.047 | *F* =0.426, *P* =0.667 | *F* =0.201, *P* =0.872 | *F* =0.624, *P* =0.548 | *F* =4.225^*^, *P* =0.026 |
| *Castanopsis hystrix* | Control | 8.16 ± 0.18^aA^ | 1.02 ± 0.06^aB^ | 10.22 ± 1.80^aD^ | 100.13 ± 2.60^aA^ | 112.01 ± 4.62^aA^ |
|  | MN | 7.60 ± 0.31^aA^ | 0.92 ± 0.06^aB^ | 9.61 ± 0.38^aC^ | 95.56 ± 5.49^aA^ | 117.60 ± 3.78^aA^ |
|  | LN | 5.77 ± 0.44^bA^ | 0.78 ± 0.02^bB^ | 8.63 ± 0.19^bC^ | 90.21 ± 2.69^aA^ | 103.46 ± 4.24^aA^ |
|  | *F* and *P* | *F* =14.559^***^, *P* <0.001 | *F* =10.168^**^, *P* =0.001 | *F* =9.204^**^, *P* =0.002 | *F* =1.673, *P* =0.216 | *F* =0.970, *P* =0.398 |
| *Betula alnoides* | Control | 8.55 ± 0.60^aA^ | 1.03 ± 0.09^aB^ | 15.37 ± 1.04^aC^ | 67.60 ± 5.45^aB^ | 120.54 ± 5.18^aA^ |
|  | MN | 8.42 ± 0.61^aA^ | 1.05 ± 0.03^aB^ | 14.32 ± 0.32^aB^ | 73.35 ± 2.38^aB^ | 112.28 ± 3.08^aA^ |
|  | LN | 4.73 ± 0.25^bA^ | 0.76 ± 0.05^bB^ | 12.63 ± 0.31^bB^ | 60.70 ± 4.88^aB^ | 89.60 ± 3.92^bA^ |
|  | *F* and *P* | *F* =17.972^***^, *P* <0.001 | *F* =6.048^**^, *P* =0.010 | *F* =4.450^*^, *P* =0.028 | *F* =2.035, *P* =0.160 | *F* =3.766^*^, *P* =0.040 |

Statistical differences between each character of different species under three N treatments (Mean ± SE) results of a one-way analysis of variance (ANOVA) (n=7). The lower case letters indicate significant differences at 0.05 levels between different N treatments, and the upper case letters indicate significant differences at 0.05 levels between the species under the same N treatment. The *F*-ratios with statistically significant values are denoted by ^*^*P*<0.05, ^**^*P*<0.01, ^***^*P*<0.001 between the three N treatments. Control, high N; MN, medium N; LN, low N.

**Table S2** Stomatal conductance (*g*_s_), mesophyll conductance (*g*_m_), CO_2_ concentration at sub-stomatal cavities (*C*_i_), CO_2_ concentration at carboxylation site (*C*_c_), and *C*_i_−*C*_c_ in the seedling leaves of the four tree species exposed to different soil nitrogen (N) treatments.

| Tree species | N treatments | *g*_s_ (mol CO_2_ m^–2^ s^–1^) | *g*_m_ (mol CO_2_ m^–2^ s^–1^) | *C*_i_ (μmol mol^-1^) | *C*_c_ (μmol mol^-1^) | *C*_i_−*C*_c_ (μmol mol^-1^) |
| --- | --- | --- | --- | --- | --- | --- |
| *Dalbergia Odorifera* | Control | 0.067 ± 0.004^aBC^ | 0.137±0.010^aA^ | 251.54 ± 6.44^bBC^ | 190.77±6.92^aB^ | 60.77±2.21^cC^ |
|  | MN | 0.066 ± 0.008^aB^ | 0.076±0.007^bB^ | 282.96 ± 4.75^aB^ | 199.54±2.10^aB^ | 83.42±3.57^bBC^ |
|  | LN | 0.053 ± 0.008^aB^ | 0.061±0.008^bB^ | 267.27 ± 10.44^abB^ | 172.31±10.67^bB^ | 94.97±4.41^aABC^ |
|  | *F* and *P* | *F* =1.072, *P* =0.372 | *F* =23.223^***^, *P* =0.001 | *F* =4.281^*^, *P* =0.030 | *F* =3.492^*^, *P* =0.039 | *F* =24.474^***^, *P* <0.001 |
| *Erythrophleum fordii* | Control | 0.046 ± 0.002^abC^ | 0.066±0.007^aC^ | 235.61 ± 6.19^aC^ | 132.59±6.90^aD^ | 103.02±4.83^aA^ |
|  | MN | 0.052 ± 0.003^aB^ | 0.071±0.004^aB^ | 245.97 ± 3.06^aC^ | 148.57±4.65^aC^ | 97.41±3.72^aAB^ |
|  | LN | 0.039 ± 0.019^bB^ | 0.064±0.004^aB^ | 234.49 ± 6.33^aC^ | 145.08±9.57^aC^ | 89.42±6.85^aBC^ |
|  | *F* and *P* | *F* =5.374^*^, *P* =0.015 | *F* =0.544, *P* =0.589 | *F* =1.237, *P* =0.314 | *F* =1.316, *P* =0.293 | *F* =1.667, *P* =0.217 |
| *Castanopsis hystrix* | Control | 0.074 ± 0.004^aB^ | 0.099±0.006^aB^ | 256.78 ± 5.24^bB^ | 167.99±6.04^aC^ | 88.79±6.26^bB^ |
|  | MN | 0.063 ± 0.005^abB^ | 0.089±0.006^aB^ | 252.27 ± 4.59^bC^ | 163.26±5.34^aC^ | 89.01±3.48^bABC^ |
|  | LN | 0.056 ± 0.005^bB^ | 0.055±0.004^bB^ | 271.26 ± 6.73^aB^ | 168.70±9.86^aBC^ | 103.56±3.73^aAB^ |
|  | *F* and *P* | *F* =4.237^*^, *P* =0.029 | *F* =16.805^***^, *P* <0.001 | *F* =4.316^*^, *P* =0.029 | *F* =0.161, *P* =0.852 | *F* =4.258^*^, *P* =0.031 |
| *Betula alnoides* | Control | 0.100 ± 0.013^aA^ | 0.134±0.012^aA^ | 292.88 ± 5.94^bA^ | 226.42±9.57^bA^ | 66.45±4.64^aC^ |
|  | MN | 0.112 ± 0.008^aA^ | 0.127±0.007^aA^ | 299.60 ± 7.88^bA^ | 230.41±10.47^bA^ | 69.20±3.33^aD^ |
|  | LN | 0.107 ± 0.010^aA^ | 0.083±0.005^bA^ | 334.44 ± 2.52^aA^ | 275.53±4.61^aA^ | 58.91±6.07^aD^ |
|  | *F* and *P* | *F* =0.168, *P* =0.851 | *F* =10.975^**^, *P* =0.001 | *F* =14.395^***^, *P* <0.001 | *F* =10.029^**^, *P* =0.001 | *F* =1.227, *P* =0.373 |

Statistical differences between each character of different species under three N treatments (Mean ± SE) results of a one-way analysis of variance (ANOVA) (n=7). Data of CO_2_ conductance was measured in light saturated and leaf chamber CO_2_ concentration was 380 μmol mol^–1^. The lower case letters indicate significant differences at 0.05 levels between different N treatments, and the upper case letters indicate significant differences at 0.05 levels between the species under the same N treatment. The *F*-ratios with statistically significant values are denoted by ^*^*P*<0.05, ^**^*P*<0.01, ^***^*P*<0.001 between the three N treatments. Control, high N; MN, medium N; LN, low N.

**Table S3** Maximum carboxylation rate (*V*_cmax_), and maximum electron transport rate (*J*_max_) in the seedling leaves of the four tree species exposed to different soil nitrogen (N) treatments.

| Tree species | N treatments | *V*_cmax_ (μmol m^–2^ s^–1^) | *J*_max_ (μmol m^–2^ s^–1^) |
| --- | --- | --- | --- |
| *Dalbergia odorifera* | Control | 78.14±4.59^aB^ | 100.71±5.80^aBC^ |
|  | MN | 77.19±3.22^aB^ | 97.81±4.17^aB^ |
|  | LN | 83.06±4.60^aA^ | 106.77±2.96^aA^ |
|  | *F* and *P* | *F* =0.567, *P* =0.577 | *F* =1.048, *P* =0.371 |
| *Erythrophleum fordii* | Control | 99.84±9.37^aA^ | 128.76±11.20^aAB^ |
|  | MN | 95.39±5.80^aA^ | 124.73±5.17^aA^ |
|  | LN | 88.21±7.05^aA^ | 116.99±9.32^aA^ |
|  | *F* and *P* | *F* =0.734, *P* =0.494 | *F* =0.495, *P* =0.618 |
| *Castanopsis hystrix* | Control | 82.78±4.47^aB^ | 109.28±3.40^aABC^ |
|  | MN | 83.04±2.71^aB^ | 105.11±4.04^aB^ |
|  | LN | 57.54±6.27^bB^ | 67.65±2.62^bB^ |
|  | *F* and *P* | *F* =13.201^***^, *P* <0.001 | *F* =45.444^***^, *P* <0.001 |
| *Betula alnoides* | Control | 72.98±3.51^aB^ | 98.38±5.37^aBC^ |
|  | MN | 74.76±3.85^aB^ | 102.16±7.66^aB^ |
|  | LN | 41.07±4.22^bC^ | 55.41±4.16^bB^ |
|  | *F* and *P* | *F* =58.030^***^, *P* <0.001 | *F* =21.933^***^, *P* <0.001 |

Statistical differences between each character of different species under three N treatments (Mean ± SE) results of a one-way analysis of variance (ANOVA) (n=7). The lower case letters indicate significant differences at 0.05 levels between different N treatments, and the upper case letters indicate significant differences at 0.05 levels between the species under the same N treatment. The *F*-ratios with statistically significant values are denoted by ^*^*P*<0.05, ^**^*P*<0.01, ^***^*P*<0.001 between the three N treatments. Control, high N; MN, medium N; LN, low N.

**Table S4** Nitrogen (N) allocation proportion of Rubisco (*P*_R_), bioenergetics (*P*_B_), light-harvesting components(*P*_L_), photosynthetic system (*P*_P_), cell wall (*P*_CW_), and other parts (*P*_Other_) in the seedling leaves of the four tree species exposed to different soil N treatments.

| Tree species | N treatments | *P*_R_ (g g^-1^) | *P*_B_ (g g^-1^) | *P*_L_ (g g^-1^) | *P*_P_ (g g^-1^) | *P*_CW_ (g g^-1^) | *P*_Other_ (g g^-1^) |
| --- | --- | --- | --- | --- | --- | --- | --- |
| *Dalbergia odorifera* | Control | 0.13±0.009^aB^ | 0.03±0.002^aB^ | 0.10±0.008^aA^ | 0.27±0.016^aB^ | 0.07±0.004^bC^ | 0.66±0.015^aA^ |
|  | MN | 0.13±0.013^aD^ | 0.03±0.004^aB^ | 0.09±0.008^aB^ | 0.25±0.022^aB^ | 0.10±0.008^aC^ | 0.65±0.018^aA^ |
|  | LN | 0.11±0.011^aA^ | 0.04±0.006^aA^ | 0.10±0.008^aA^ | 0.25±0.022^aC^ | 0.12±0.012^aC^ | 0.63±0.030^aA^ |
|  | *F* and *P* | *F* =1.100, *P* =0.354 | *F* =0.819, *P* =0.457 | *F* =1.045, *P* =0.372 | *F* =0.350, *P* =0.709 | *F* =8.692^**^, *P* =0.002 | *F* =0.424, *P* =0.661 |
| *Erythrophleum fordii* | Control | 0.16±0.010^aB^ | 0.04±0.003^aB^ | 0.06±0.009^bB^ | 0.27±0.018^aB^ | 0.05±0.002^aC^ | 0.68±0.019^aA^ |
|  | MN | 0.18±0.015^aC^ | 0.05±0.003^aB^ | 0.09±0.004^aB^ | 0.31±0.017^aB^ | 0.06±0.006^aD^ | 0.63±0.020^aA^ |
|  | LN | 0.18±0.013^aB^ | 0.04±0.003^aA^ | 0.08±0.006^aB^ | 0.30±0.021^aB^ | 0.05±0.003^aD^ | 0.65±0.020^aA^ |
|  | *F* and *P* | *F* =0.629, *P* =0.545 | *F* =2.139, *P* =0.147 | *F* =4.569^*^, *P* =0.025 | *F* =1.740, *P* =0.204 | *F* =0.766, *P* =0.479 | *F* =1.804, *P* =0.193 |
| *Castanopsis hystrix* | Control | 0.30±0.012^aA^ | 0.07±0.003^aA^ | 0.07±0.008^aB^ | 0.44±0.018^aA^ | 0.27±0.010^bA^ | 0.29±0.019^aB^ |
|  | MN | 0.29±0.017^aA^ | 0.07±0.003^aA^ | 0.07±0.007^aC^ | 0.42±0.023^aA^ | 0.46±0.027^aA^ | 0.12±0.012^cC^ |
|  | LN | 0.26±0.025^aA^ | 0.05±0.004^bA^ | 0.07±0.008^aB^ | 0.38±0.030^aA^ | 0.45±0.025^aA^ | 0.17±0.015^bC^ |
|  | *F* | *F* =1.190, *P* =0.327 | *F* =7.734^**^, *P* =0.004 | *F* =0.123, *P* =0.885 | *F* =1.482, *P* =0.254 | *F* =23.426^***^, *P* <0.001 | *F* =29.593^***^, *P* <0.001 |
| *Betula alnoides* | Control | 0.26±0.028^aA^ | 0.07±0.007^aA^ | 0.12±0.011^aA^ | 0.44±0.042^aA^ | 0.22±0.011^bB^ | 0.34±0.042^aB^ |
|  | MN | 0.23±0.019^aB^ | 0.06±0.006^aA^ | 0.11±0.005^aA^ | 0.40±0.027^aA^ | 0.28±0.023^aB^ | 0.32±0.045^aB^ |
|  | LN | 0.16±0.011^bB^ | 0.04±0.001^bA^ | 0.08±0.007^bB^ | 0.29±0.009^bB^ | 0.33±0.016^aB^ | 0.39±0.013^aB^ |
|  | *F* and *P* | *F* =5.430^*^, *P* =0.014 | *F* =6.143^**^, *P* =0.009 | *F* =4.088^*^, *P* =0.034 | *F* =4.796^*^, *P* =0.021 | *F* =9.389^**^, *P* =0.002 | *F* =0.106, *P* =0.900 |

Statistical differences between each character of different species under three N treatments (Mean ± SE) results of a one-way analysis of variance (ANOVA) (n=7). The lower case letters indicate significant differences at 0.05 levels between different N treatments, and the upper case letters indicate significant differences at 0.05 levels between the species under the same N treatment. The *F*-ratios with statistically significant values are denoted by ^*^*P*<0.05, ^**^*P*<0.01, ^***^*P*<0.001 between the three N treatments. Control, high N; MN, medium N; LN, low N.

**Table S5** Varieties between each functional group (N-fixing and non-N-fixing) under different soil nitrogen (N) treatments.

| Group | N treatments | *A*_max_′ (μmol m^–2^ s^–1^) | *N*_area_ (g m^-2^) | *N*_mass_ (mg g^-1^) | LMA (g m^-2^) | PNUE (μmol mol^–1^ s^–1^) | *g*_s_ (mol CO_2_ m^–2^ s^–1^) | *g*_m_ (mol CO_2_ m^–2^ s^–1^) |
| --- | --- | --- | --- | --- | --- | --- | --- | --- |
| N-fixing | Control | 7.32±0.38^aB^ | 2.10±0.09^aA^ | 0.030±0.001^aA^ | 70.16±1.95^aB^ | 3.52±0.17^aB^ | 0.05±0.004^aB^ | 0.10±0.011^aA^ |
|  | MN | 6.56±0.33^abB^ | 2.03±0.08^aA^ | 0.029±0.001^abA^ | 70.57±1.76^aB^ | 3.25±0.13^aB^ | 0.06±0.004^aB^ | 0.07±0.004^bB^ |
|  | LN | 5.49±0.34^bA^ | 1.97±0.08^aA^ | 0.026±0.001^bA^ | 75.86±2.28^aA^ | 2.97±0.19^aB^ | 0.05±0.004^aB^ | 0.06±0.005^bA^ |
|  | *F* and *P* | *F*=6.765^**^, *P*=0.003 | *F*=0.521, *P*=0.598 | *F*=3.977^*^, *P*=0.027 | *F*=2.504, *P*=0.095 | *F*=2.764, *P*=0.075 | *F*=2.436, *P*=0.101 | *F*=7.277^**^, *P*=0.002 |
| non-N-fixing | Control | 8.36±0.31^aA^ | 1.03±0.05^aB^ | 0.013±0.001^aB^ | 83.86±5.36^aA^ | 8.31±0.38^aA^ | 0.09±0.008^aA^ | 0.12±0.008^aA^ |
|  | MN | 8.02±0.31^aA^ | 0.98±0.04^aB^ | 0.012±0.001^aB^ | 84.46±4.21^aA^ | 8.21±0.26^aA^ | 0.09±0.008^aA^ | 0.11±0.007^aA^ |
|  | LN | 5.25±0.28^bA^ | 0.77±0.03^bB^ | 0.011±0.001^bB^ | 75.46±4.89^aA^ | 6.95±0.44^bA^ | 0.08±0.009^aA^ | 0.07±0.005^bA^ |
|  | *F* and *P* | *F*=31.959^***^, *P*<0.001 | *F*=13.205^***^, *P*<0.001 | *F*=4.315^*^, *P*=0.022 | *F*=1.079, *P*=0.350 | *F*=4.173^*^, *P*=0.023 | *F*=0.226, *P*=0.799 | *F*=13.767^***^, *P*<0.001 |
| Group | N treatments | *C*_i_ (μmol mol^-1^) | *C*_c_ (μmol mol^-1^) | *C*_i_−*C*_c_ (μmol mol^-1^) | *V*_cmax_ (μmol m^–2^ s^–1^) | *J*_max_ (μmol m^–2^ s^–1^) | *P*_R_ (g g^-1^) | *P*_B_ (g g^-1^) |
| N-fixing | Control | 243.58±5.05^aB^ | 161.68±9.33^aB^ | 81.89±6.39^aA^ | 88.99±5.85^aA^ | 114.74±7.20^aA^ | 0.15±0.008^aB^ | 0.04±0.002^aB^ |
|  | MN | 264.47±5.80^aA^ | 174.06±7.48^aA^ | 90.41±3.15^aA^ | 86.29±3.08^aA^ | 111.27±4.36^aA^ | 0.15±0.011^aB^ | 0.04±0.002^aB^ |
|  | LN | 250.88±7.42^aB^ | 158.69±7.85^aB^ | 92.19±3.99^aA^ | 85.63±4.11^aB^ | 111.88±4.90^aA^ | 0.15±0.013^aB^ | 0.04±0.003^aA^ |
|  | *F* and *P* | *F*=2.953, *P*=0.064 | *F*=0.972, *P*=0.387 | *F*=1.364, *P*=0.267 | *F*=0.156, *P*=0.856 | *F*=0.108, *P*=0.898 | *F*=0.068, *P*=0.934 | *F*=1.380, *P*=0.264 |
| non-N-fixing | Control | 274.83±6.29^bA^ | 197.21±9.76^aA^ | 77.62±4.86^aA^ | 77.88±1.69^aA^ | 103.83±3.41^aA^ | 0.28±0.016^aA^ | 0.07±0.004^aA^ |
|  | MN | 275.94±7.89^bA^ | 197.42±10.92^aA^ | 78.52±3.69^aB^ | 78.90±2.54^aA^ | 103.63±4.18^aA^ | 0.26±0.014^aA^ | 0.06±0.003^aA^ |
|  | LN | 304.35±9.03^aA^ | 222.12±15.71^aA^ | 82.23±7.32^aA^ | 49.30±3.82^bB^ | 61.53±2.35^bB^ | 0.21±0.019^bA^ | 0.05±0.002^bA^ |
|  | *F* and *P* | *F*=4.581^*^, *P*=0.016 | *F*=1.334, *P*=0.275 | *F*=0.198, *P*=0.822 | *F*=35.396^***^, *P*<0.001 | *F*=51.438^***^, *P*<0.001 | *F*=4.254^*^, *P*=0.021 | *F*=12.350^***^, *P*<0.001 |

| Group | | N treatments | | *P*_L_ (g g^-1^) | | *P*_P_ (g g^-1^) | *P*_CW_ (g g^-1^) | *P*_Other_ (g g^-1^) | *g*_m_(molCO_2_ m^–2^ s^–1^) Harley | *g*_m_(molCO_2_ m^–2^ s^–1^)Ethier | *g*_m_(molCO_2_ m^–2^ s^–1^) Gu |
| --- | --- | --- | --- | --- | --- | --- | --- | --- | --- | --- | --- |
| N-fixing | | Control | | 0.08±0.009^aA^ | | 0.27±0.012^aB^ | 0.06±0.003^aB^ | 0.67±0.012^aA^ | 0.09±0.010^aB^ | 0.10±0.012^aA^ | 0.11±0.014^aA^ |
|  |  | MN | | 0.09±0.005^aA^ | | 0.27±0.016^aB^ | 0.07±0.008^aB^ | 0.65±0.013^aA^ | 0.07±0.005^bB^ | 0.07±0.004^bB^ | 0.08±0.004^bB^ |
|  |  | LN | | 0.09±0.005^aA^ | | 0.28±0.016^aB^ | 0.09±0.011^aB^ | 0.63±0.017^aA^ | 0.06±0.005^bA^ | 0.06±0.005^bA^ | 0.07±0.005^bA^ |
|  |  | *F* and *P* | | *F*=0.776, *P*=0.467 | | *F*=0.208, *P*=0.813 | *F*=2.917, *P*=0.066 | *F*=2.006, *P*=0.148 | *F*=7.449^**^, *P*=0.002 | *F*=7.506^**^, *P*=0.002 | *F*=6.365^**^, *P*=0.004 |
| non-N-fixing | | Control | | 0.09±0.009^aA^ | | 0.44±0.022^aA^ | 0.24±0.010^bA^ | 0.32±0.023^aB^ | 0.12±0.008^aA^ | 0.11±0.006^aA^ | 0.11±0.011^aA^ |
|  |  | MN | | 0.08±0.006^aA^ | | 0.40±0.018^aA^ | 0.37±0.030^aA^ | 0.23±0.038^aB^ | 0.11±0.007^aA^ | 0.11±0.007^aA^ | 0.11±0.007^aA^ |
|  |  | LN | | 0.09±0.007^aA^ | | 0.35±0.018^bA^ | 0.39±0.022^aA^ | 0.27±0.028^aB^ | 0.07±0.005^bA^ | 0.07±0.005^bA^ | 0.07±0.005^bA^ |
|  |  | *F* and *P* | | *F*=1.744, *P*=0.188 | | *F*=5.803^**^, *P*=0.006 | *F*=12.240^***^, *P*<0.001 | *F*=1.833, *P*=0.173 | *F*=18.648^***^, *P*<0.001 | *F*=13.299^***^, *P*<0.001 | *F*=7.198^**^, *P*=0.002 |
| Group | | N treatments | | *C*_c_(μmol mol^–1^) Harley | | *C*_c_(μmol mol^–1^) Ethier | *C*_c_(μmol mol^–1^) Gu | *C*_mass_ (mg g^-1^) | *C/N* (g g^–1^) | *Chl*a(mg g^-1^) | *Chl*b(mg g^-1^) |
| N-fixing | | Control | | 157.60±7.32^aB^ | | 163.28±9.70^aB^ | 164.17±11.64^aA^ | 483.07±12.25^aA^ | 16.47±0.83^aB^ | 1.04±0.10^aA^ | 0.81±0.08^bA^ |
|  |  | MN | | 166.61±7.22^aA^ | | 175.46±7.56^aA^ | 180.11±8.16^aA^ | 496.97±9.96^aA^ | 17.58±0.70^aB^ | 1.05±0.06^aA^ | 0.81±0.05^bA^ |
|  |  | LN | | 147.76±7.81^aB^ | | 161.24±7.87^aB^ | 167.08±9.43^aB^ | 484.03±8.39^aA^ | 18.81±0.61^aB^ | 1.17±0.05^aA^ | 1.08±0.07^aA^ |
|  |  | *F* and *P* | | *F*=1.598, *P*=0.215 | | *F*=0.832, *P*=0.443 | *F*=0.743, *P*=0.482 | *F*=0.566, *P*=0.573 | *F*=2.652, *P*=0.083 | *F*=1.020, *P*=0.370 | *F*=5.167^*^, *P*=0.010 |
| non-N-fixing | | Control | | 200.48±9.31^aA^ | | 199.42±8.51^aA^ | 191.72±12.00^aA^ | 486.64±3.94^aA^ | 40.00±2.27^aA^ | 0.64±0.08^aB^ | 0.52±0.05^aB^ |
|  | MN | | 191.70±11.53^aA^ | | 200.89±10.50^aA^ | | 199.65±10.89^aA^ | 497.60±6.24^aA^ | 43.66±2.80^aA^ | 0.58±0.06^aB^ | 0.46±0.04^aB^ |
|  | LN | | 217.18±15.68^aA^ | | 225.19±16.03^aA^ | | 223.98±15.75^aA^ | 479.52±5.85^aA^ | 46.82±2.49^aA^ | 0.59±0.07^aB^ | 0.45±0.05^aB^ |
|  | *F* and *P* | | *F*=1.080, *P*=0.350 | | *F*=1.428, *P*=0.252 | | *F*=1.660, *P*=0.203 | *F*=2.808, *P*=0.073 | *F*=1.821, *P*=0.175 | *F*=0.216, *P*=0.807 | *F*=0.621, *P*=0.543 |

| Group | N treatments | *Chl*a+b(mg g^-1^) | *Q*_Rarea_(g·m^-2^) | *Q*_Barea_(g·m^-2^) | *Q*_Larea_(g·m^-2^) | *Q*_Parea_(g·m^-2^) | *Q*_CWarea_(g·m^-2^) | *Q*_Other-area_(g·m^-2^) |
| --- | --- | --- | --- | --- | --- | --- | --- | --- |
| N-fixing | Control | 1.85±0.18^aA^ | 0.31±0.02^aA^ | 0.08±0.005^aA^ | 0.17±0.018^aA^ | 0.55±0.02^aA^ | 0.13±0.01^aB^ | 1.42±0.07^aA^ |
|  | MN | 2.10±0.10^aA^ | 0.31±0.02^aA^ | 0.07±0.004^aA^ | 0.17±0.011^aA^ | 0.55±0.03^aA^ | 0.15±0.02^aB^ | 1.32±0.06^aA^ |
|  | LN | 2.01±0.13^aA^ | 0.29±0.02^aA^ | 0.08±0.006^aA^ | 0.18±0.012^aA^ | 0.55±0.03^aA^ | 0.17±0.02^aB^ | 1.26±0.08^aA^ |
|  | *F* and *P* | *F*=0.846, *P*=0.437 | *F*=0.420, *P*=0.660 | *F*=1.257, *P*=0.296 | *F*=0.168, *P*=0.846 | *F*=0.010, *P*=0.990 | *F*=1.549, *P*=0.225 | *F*=1.264, *P*=0.294 |
| non-N-fixing | Control | 1.16±0.14^aB^ | 0.28±0.01^aA^ | 0.07±0.002^aA^ | 0.09±0.008^aB^ | 0.44±0.01^aB^ | 0.25±0.02^bA^ | 0.34±0.04^aB^ |
|  | MN | 1.04±0.10^aB^ | 0.25±0.01^aB^ | 0.06±0.004^aA^ | 0.09±0.009^aB^ | 0.40±0.02^aB^ | 0.36±0.03^aA^ | 0.22±0.04^bB^ |
|  | LN | 1.03±0.12^aB^ | 0.16±0.01^bB^ | 0.04±0.002^bB^ | 0.06±0.006^bB^ | 0.26±0.02^bB^ | 0.30±0.02^aA^ | 0.21±0.03^bB^ |
|  | *F* and *P* | *F*=0.341, *P*=0.713 | *F*=25.065^***^, *P*<0.001 | *F*=34.836^***^, *P*<0.001 | *F*=4.997^*^, *P*=0.012 | *F*=37.608^***^, *P*<0.001 | *F*=5.416^**^, *P*=0.008 | *F*=4.227^*^, *P*=0.022 |
| Group | N treatments | *Q*_Rmass_(mg·g^-1^) | *Q*_Bmass_(mg·g^-1^) | *Q*_Lmass_(mg·g^-1^) | *Q P*_mass_(mg·g^-1^) | *Q*_CWmass_(mg·g^-1^) | *Q*_Other-mass_(mg·g^-1^) |  |
| N-fixing | Control | 4.43±0.23^aA^ | 1.07±0.06^aA^ | 2.48±0.29^aA^ | 7.97±0.38^aA^ | 1.80±0.13^aA^ | 20.12±0.78^aA^ |  |
|  | MN | 4.40±0.36^aA^ | 1.01±0.07^aA^ | 2.48±0.18^aA^ | 7.89±0.55^aA^ | 2.14±0.23^aA^ | 18.72±0.73^abA^ |  |
|  | LN | 3.84±0.38^aA^ | 1.06±0.07^aA^ | 2.41±0.12^aA^ | 7.30±0.46^aA^ | 2.18±0.22^aA^ | 16.57±0.82^bA^ |  |
|  | *F* and *P* | *F*=1.021, *P*=0.370 | *F*=0.218, *P*=0.805 | *F*=0.040, *P*=0.961 | *F*=0.604, *P*=0.552 | *F*=1.110, *P*=0.340 | *F*=5.297^**^, *P*=0.009 |  |
| non-N-fixing | Control | 3.47±0.20^aB^ | 0.84±0.07^aB^ | 1.23±0.15^aB^ | 5.54±0.37^aB^ | 3.06±0.17^bB^ | 4.20±0.55^aB^ |  |
|  | MN | 3.03±0.19^aB^ | 0.76±0.06^aB^ | 1.08±0.14^aB^ | 4.87±0.34^aB^ | 4.20±0.22^aB^ | 2.89±0.54^aB^ |  |
|  | LN | 2.17±0.14^bB^ | 0.48±0.02^bB^ | 0.83±0.08^aB^ | 3.48±0.17^bB^ | 3.98±0.14^aB^ | 3.18±0.49aB |  |
|  | *F* and *P* | *F*=13.449^***^, *P*<0.001 | *F*=12.969^***^, *P*<0.001 | *F*=2.563, *P*=0.090 | *F*=11.773^***^, *P*<0.001 | *F*=11.483^***^, *P*<0.001 | *F*=1.709, *P*=0.194 |  |

Statistical differences between each character of four species under different N treatments (Means±SE) results of a one-way ANOVA (n=14). Lower case letters indicate significant difference at 0.05 levels between N treatment and capital letter indicate significant difference at 0.05 levels between species under same N treatment. *F*-ratios with statistically significant values denoted by ^*^*P*<0.05, ^**^*P*<0.01, ^***^*P*<0.001 between N treatment. Control, high N; MN, medium N; LN, low N.**Table S6** Quantity of leaf N per area allocated to Rubisco (*Q*_Rarea_), bioenergetics (*Q*_Barea_), light-harvesting components (*Q*_Larea_), photosynthetic apparatus (*Q*_Parea_), cell wall (*Q*_CWarea_), and other parts (*Q*_Other-area_) in four species seedling leaves under different soil nitrogen (N) treatments

| Tree species | N treatments | *Q*_Rarea_(g·m^-2^) | *Q*_Barea_(g·m^-2^) | *Q*_Larea_(g·m^-2^) | *Q*_Parea_(g·m^-2^) | *Q*_CWarea_(g·m^-2^) | *Q*_Other-area_(g·m^-2^) |
| --- | --- | --- | --- | --- | --- | --- | --- |
| *Dalbergia Odorifera* | Control | 0.29±0.01^aAB^ | 0.06±0.00^aB^ | 0.22±0.01^aA^ | 0.58±0.02^aA^ | 0.15±0.01^bB^ | 1.46±0.11^aA^ |
|  | MN | 0.27±0.04^aAB^ | 0.07±0.01^aA^ | 0.19±0.02^aA^ | 0.52±0.06^aAB^ | 0.21±0.02^abB^ | 1.39±0.12^aA^ |
|  | LN | 0.23±0.02^aB^ | 0.07±0.01^aA^ | 0.20±0.01^aA^ | 0.51±0.04^aA^ | 0.24±0.02^aB^ | 1.33±0.12^aA^ |
|  | *F* and *P* | *F*=1.318, *P*=0.292 | *F*=0.629, *P*=0.544 | *F*=1.475, *P*=0.255 | *F*=0.721, *P*=0.500 | *F*=7.337^**^, *P*=0.005 | *F*=0.323, *P*=0.728 |
| *Erythrophleum fordii* | Control | 0.33±0.03^aA^ | 0.09±0.01^aA^ | 0.12±0.02^aB^ | 0.53±0.04^aAB^ | 0.10±0.01^aB^ | 1.37±0.10^aA^ |
|  | MN | 0.35±0.02^aA^ | 0.07±0.01^aA^ | 0.16±0.01^aA^ | 0.58±0.04^aA^ | 0.10±0.01^aC^ | 1.26±0.06^aA^ |
|  | LN | 0.34±0.03^aA^ | 0.08±0.01^aA^ | 0.16±0.02^aA^ | 0.59±0.04^aA^ | 0.10±0.01^aC^ | 1.18±0.10^aA^ |
|  | *F* and *P* | *F*=0.091, *P*=0.913 | *F*=1.524, *P*=0.245 | *F*=2.782, *P*=0.089 | *F*=0.505, *P*=0.612 | *F*=0.272, *P*=0.765 | *F*=1.202, *P*=0.324 |
| *Castanopsis hystrix* | Control | 0.31±0.01^aAB^ | 0.07±0.00^aAB^ | 0.07±0.01^aB^ | 0.45±0.02^aBC^ | 0.27±0.01^bA^ | 0.30±0.02^aB^ |
|  | MN | 0.26±0.02^bAB^ | 0.06±0.00^aA^ | 0.06±0.01^aC^ | 0.38±0.02^bB^ | 0.42±0.04^aA^ | 0.12±0.02^bB^ |
|  | LN | 0.20±0.02^cB^ | 0.04±0.00^bB^ | 0.05±0.01^aB^ | 0.29±0.02^cB^ | 0.35±0.03^abA^ | 0.14±0.02^bB^ |
|  | *F* and *P* | *F*=15.458^***^, *P*<0.001 | *F*=28.101^***^, *P*<0.001 | *F*=2.046, *P*=0.158 | *F*=18.191^***^, *P*<0.001 | *F*=6.540^**^, *P*=0.007 | *F*=30.455^***^, *P*<0.001 |
| *Betula alnoides* | Control | 0.25±0.01^aB^ | 0.06±0.00^aB^ | 0.12±0.01^aB^ | 0.43±0.01^aC^ | 0.23±0.03^aA^ | 0.37±0.07^aB^ |
|  | MN | 0.24±0.02^aB^ | 0.07±0.01^aA^ | 0.11±0.01^aB^ | 0.42±0.03^aAB^ | 0.29±0.03^aB^ | 0.34±0.04^aB^ |
|  | LN | 0.12±0.01^bC^ | 0.03±0.00^bB^ | 0.07±0.01^bB^ | 0.22±0.02^bB^ | 0.25±0.03^aB^ | 0.29±0.02^aB^ |
|  | *F* and *P* | *F*=22.896^***^, *P*<0.001 | *F*=16.684^***^, *P*<0.001 | *F*=10.936^**^, *P*=0.001 | *F*=28.861^***^, *P*<0.001 | *F*=1.398, *P*=0.273 | *F*=0.743, *P*=0.490 |

Statistical differences between each character of four species under different N treatments (Means±SE) results of a one-way ANOVA (n=7). Lower case letters indicate significant difference at 0.05 levels between N treatment and capital letter indicate significant difference at 0.05 levels between species under same N treatment. *F*-ratios with statistically significant values denoted by ^*^*P*<0.05, ^**^*P*<0.01, ^***^*P*<0.001 between N treatment. Control, high N; MN, medium N; LN, low N.

**Table S7** Quantity of leaf N per mass allocated to Rubisco (*Q*_Rmass_), bioenergetics (*Q*_Bmass_), light-harvesting components (*Q*_Lmass_), photosynthetic apparatus (*Q*_Pmass_), cell wall (*Q*_CWmass_), and other parts (*Q*_Other-mass_) in four species seedling leaves under different soil nitrogen (N) treatments

| Tree species | N treatments | *Q*_Rmass_(mg·g^-1^) | *Q*_Bmass_(mg·g^-1^) | *Q*_Lmass_(mg·g^-1^) | *Q*_Pmass_(mg·g^-1^) | *Q*_CWmass_(mg·g^-1^) | *Q*_Other-mass_(mg·g^-1^) |
| --- | --- | --- | --- | --- | --- | --- | --- |
| *Dalbergia Odorifera* | Control | 4.24±0.24^aA^ | 0.94±0.03^aAB^ | 3.32±0.25^aA^ | 8.50±0.46^aA^ | 2.16±0.16^bB^ | 21.05±0.86^aA^ |
|  | MN | 3.84±0.56^abAB^ | 1.00±0.12^aA^ | 2.67±0.31^aA^ | 7.51±0.93^abA^ | 2.90±0.14^aB^ | 19.47±1.27^abA^ |
|  | LN | 2.76±0.26^bB^ | 0.90±0.10^aB^ | 2.46±0.18^aA^ | 6.12±0.42^bB^ | 2.85±0.18^aB^ | 16.07±1.41^bA^ |
|  | *F* and *P* | *F*=4.052^*^, *P*=0.035 | *F*=0.340, *P*=0.716 | *F*=3.100, *P*=0.070 | *F*=3.391, *P*=0.056 | *F*=6.556^**^, *P*=0.007 | *F*=4.465^*^, *P*=0.027 |
| *Erythrophleum fordii* | Control | 4.61±0.39^aA^ | 1.19±0.10^aA^ | 1.64±0.24^bB^ | 7.44±0.56^aAB^ | 1.44±0.06^aC^ | 19.20±1.27^aA^ |
|  | MN | 4.96±0.40^aA^ | 1.01±0.09^aA^ | 2.30±0.19^aA^ | 8.27±0.63^aA^ | 1.38±0.10^aC^ | 17.97±0.70^aA^ |
|  | LN | 4.91±0.40^aA^ | 1.22±0.08^aA^ | 2.36±0.18^aA^ | 8.48±0.53^aA^ | 1.50±0.14^aC^ | 17.08±0.92^aA^ |
|  | *F* and *P* | *F*=0.231, *P*=0.796 | *F*=1.408, *P*=0.270 | *F*=3.841^*^, *P*=0.041 | *F*=0.918, *P*=0.417 | *F*=0.342, *P*=0.715 | *F*=1.159, *P*=0.336 |
| *Castanopsis hystrix* | Control | 3.08±0.10^aB^ | 0.69±0.04^aB^ | 0.73±0.09^aC^ | 4.51±0.18^aC^ | 2.73±0.11^bAB^ | 2.99±0.22^aB^ |
|  | MN | 2.75±0.22^abB^ | 0.63±0.05^aB^ | 0.65±0.09^aC^ | 4.02±0.33^abB^ | 4.39±0.24^aA^ | 1.20±0.15^bC^ |
|  | LN | 2.26±0.22^bB^ | 0.44±0.03^bC^ | 0.60±0.07^aB^ | 3.30±0.27^bC^ | 3.83±0.19^aA^ | 1.49±0.16^bC^ |
|  | *F* and *P* | *F*=4.895^*^, *P*=0.020 | *F*=11.266^**^, *P*=0.001 | *F*=0.614, *P*=0.552 | *F*=5.046^*^, *P*=0.018 | *F*=19.921^***^, *P*<0.001 | *F*=29.349^***^, *P*<0.001 |
| *Betula alnoides* | Control | 3.85±0.34^aAB^ | 0.99±0.10^aAB^ | 1.72±0.07^aB^ | 6.57±0.45^aB^ | 3.39±0.29^aA^ | 5.42±0.88^aB^ |
|  | MN | 3.31±0.29^aB^ | 0.89±0.09^aAB^ | 1.52±0.09^aB^ | 5.72±0.40^aB^ | 4.02±0.37^aA^ | 4.59±0.54^aB^ |
|  | LN | 2.08±0.19^bB^ | 0.51±0.02^bC^ | 1.06±0.09^bB^ | 3.65±0.21^bC^ | 4.12±0.19^aA^ | 4.86±0.25^aB^ |
|  | *F* and *P* | *F*=10.368^**^, *P*=0.001 | *F*=10.115^**^, *P*=0.001 | *F*=16.787^***^, *P*<0.001 | *F*=16.825^***^, *P*<0.001 | *F*=1.866, *P*=0.183 | *F*=0.475, *P*=0.630 |

Statistical differences between each character of four species under different N treatments (Means±SE) results of a one-way ANOVA (n=7). Lower case letters indicate significant difference at 0.05 levels between N treatment and capital letter indicate significant difference at 0.05 levels between species under same N treatment. *F*-ratios with statistically significant values denoted by ^*^*P*<0.05, ^**^*P*<0.01, ^***^*P*<0.001 between N treatment. Control, high N; MN, medium N; LN, low N.

**Table S8** Tests of Between-Subjects effects of varieties of trees and N treatments on variables in four species.

| Variable | *F*-value | | | *P*-value | | | Variable | *F*-value | | | *P*-value | | |
| --- | --- | --- | --- | --- | --- | --- | --- | --- | --- | --- | --- | --- | --- |
|  | V-E | N-E | V×N-E | V-E | N-E | V×N-E |  | V-E | N-E | V×N-E | V-E | N-E | V×N-E |
| *A*_max_*′* | 33.108 | 3.070 | 2.962 | <0.001 | 0.033 | 0.012 | *g*_m_ Harley | 32.645 | 19.534 | 3.243 | <0.001 | <0.001 | 0.007 |
| *N*_area_ | 4.411 | 144.485 | 0.416 | 0.016 | <0.001 | 0.866 | *g*_m_ Ethier | 35.911 | 24.376 | 7.834 | <0.001 | <0.001 | <0.001 |
| *N*_mass_ | 9.264 | 287.216 | 1.700 | <0.001 | <0.001 | 0.133 | *g*_m_ Gu | 30.596 | 25.551 | 10.006 | <0.001 | <0.001 | <0.001 |
| LMA | 0.318 | 41.899 | 3.422 | 0.729 | <0.001 | 0.005 | *C*_c_ Harley | 0.265 | 102.638 | 5.570 | 0.768 | <0.001 | <0.001 |
| PNUE | 6.399 | 131.320 | 1.737 | 0.003 | <0.001 | 0.125 | *C*_c_ Ethier | 2.329 | 91.329 | 5.366 | 0.105 | <0.001 | <0.001 |
| *g*_s_ | 2.169 | 44.766 | 0.646 | 0.122 | <0.001 | 0.693 | *C*_c_ Gu | 3.856 | 70.210 | 3.155 | 0.026 | <0.001 | 0.008 |
| *g*_m_ | 37.924 | 25.035 | 7.149 | <0.001 | <0.001 | <0.001 | *Chl*a | 1.214 | 129.203 | 3.830 | 0.303 | <0.001 | 0.002 |
| *C*_i_ | 8.811 | 66.237 | 4.927 | <0.001 | <0.001 | <0.001 | *Chl*b | 5.026 | 65.656 | 9.275 | 0.009 | <0.001 | <0.001 |
| *C*_c_ | 2.070 | 97.926 | 5.016 | 0.134 | <0.001 | <0.001 | *Chl*a+b | 0.358 | 94.575 | 7.103 | 0.700 | <0.001 | <0.001 |
| *C*_i_−*C*_c_ | 2.679 | 31.481 | 6.419 | 0.075 | <0.001 | <0.001 | *Q*_Rarea_ | 12.362 | 19.811 | 2.464 | <0.001 | <0.001 | 0.032 |
| *V*_cmax_ | 14.862 | 23.609 | 4.532 | <0.001 | <0.001 | 0.001 | *Q*_Barea_ | 6.479 | 15.310 | 5.908 | 0.003 | <0.001 | <0.001 |
| *J*_max_ | 18.707 | 23.828 | 6.220 | <0.001 | <0.001 | <0.001 | *Q*_Larea_ | 0.849 | 77.759 | 3.948 | 0.432 | <0.001 | 0.002 |
| *P*_R_ | 4.827 | 51.092 | 2.100 | 0.011 | <0.001 | 0.064 | *Q*_Parea_ | 9.375 | 32.936 | 3.905 | <0.001 | <0.001 | 0.002 |
| *P*_B_ | 4.193 | 29.791 | 4.624 | 0.019 | <0.001 | 0.001 | *Q*_CWarea_ | 9.552 | 67.116 | 2.822 | <0.001 | <0.001 | 0.016 |
| *P*_L_ | 1.811 | 13.102 | 2.535 | 0.171 | <0.001 | 0.028 | *Q*_Other-area_ | 3.616 | 200.862 | 0.227 | 0.032 | <0.001 | 0.967 |
| *P*_P_ | 2.928 | 28.596 | 2.505 | 0.060 | <0.001 | 0.029 | *Q*_Rmass_ | 9.284 | 24.962 | 2.086 | <0.001 | <0.001 | 0.065 |
| *P*_CW_ | 37.227 | 340.216 | 10.338 | <0.001 | <0.001 | <0.001 | *Q*_Bmass_ | 5.952 | 26.782 | 3.323 | 0.004 | <0.001 | 0.006 |
| *P*_Other_ | 5.121 | 258.044 | 2.982 | 0.008 | <0.001 | 0.012 | *Q*_Lmass_ | 1.920 | 85.822 | 4.696 | 0.154 | <0.001 | <0.001 |
| *C*_mass_ | 4.130 | 28.384 | 0.984 | 0.020 | <0.001 | 0.442 | *Q*_Pmass_ | 8.305 | 44.929 | 3.285 | 0.001 | <0.001 | 0.007 |
| *C/N* | 14.758 | 571.042 | 2.371 | <0.001 | <0.001 | 0.038 | *Q*_CWmass_ | 16.502 | 92.534 | 3.353 | <0.001 | <0.001 | 0.006 |
|  |  |  |  |  |  |  | *Q*_Other-mass_ | 7.470 | 326.326 | 1.604 | 0.001 | <0.001 | 0.159 |

Effects of varieties of trees and N treatments on variables in four species were analyzed by two-way ANOVA and the TUKEY test. V-E = Varieties of trees interaction effect; N-E = N treatments interaction effect; V×N-E = Varieties of trees ×N treatments interaction effect.

**Table S9** Mesophyll conductance (*g*_m_), and CO_2_ concentration at carboxylation site (*C*_c_) calculated by three methods in four species seedling leaves under different soil nitrogen (N) treatments.

| Tree species | N treatments | *g*_m_(molCO_2_ m^–2^ s^–1^) Harley | *g*_m_(molCO_2_ m^–2^ s^–1^)Ethier | *g*_m_(molCO_2_ m^–2^ s^–1^) Gu | *C*_c_(μmol mol^–1^) Harley | *C*_c_(μmol mol^–1^) Ethier | *C*_c_(μmol mol^–1^) Gu |
| --- | --- | --- | --- | --- | --- | --- | --- |
| *Dalbergia Odorifera* | Control | 0.114±0.013^aB^ | 0.140±0.011^aA^ | 0.159±0.006^aA^ | 178.39±7.84^abB^ | 192.99±7.11^abB^ | 200.92±6.72^abA^ |
|  | MN | 0.070±0.009^bB^ | 0.076±0.006b^BC^ | 0.083±0.006^bB^ | 190.80±3.14^aB^ | 200.32±2.93^aB^ | 207.52±3.26^aB^ |
|  | LN | 0.055±0.010^bB^ | 0.062±0.008^bB^ | 0.068±0.008^bB^ | 158.31±12.37^bB^ | 174.25±10.65^bB^ | 184.36±10.76^bB^ |
|  | *F* and *P* | *F*=8.357^**^, *P*=0.003 | *F*=23.471^***^, *P*<0.001 | *F*=54.850^***^, *P*<0.001 | *F*=3.597^*^, *P*=0.049 | *F*=3.140, *P*=0.068 | *F*=2.488, *P*=0.111 |
| *Erythrophleum fordii* | Control | 0.068±0.007^aC^ | 0.066±0.007^aC^ | 0.063±0.007^aB^ | 136.80±5.19^aC^ | 133.56±7.95^aC^ | 127.42±9.56^aC^ |
|  | MN | 0.067±0.004^aB^ | 0.073±0.004^aC^ | 0.074±0.005^aB^ | 142.42±4.60^aC^ | 150.60±5.76^aC^ | 152.70±5.23^aC^ |
|  | LN | 0.059±0.002^aB^ | 0.064±0.005^aB^ | 0.069±0.007^aB^ | 137.22±8.63^aB^ | 148.22±9.94^aB^ | 149.79±13.02^aB^ |
|  | *F* and *P* | *F*=1.035, *P*=0.375 | *F*=0.664, *P*=0.527 | *F*=0.884, *P*=0.430 | *F*=0.240, *P*=0.789 | *F*=1.309, *P*=0.294 | *F*=1.990, *P*=0.166 |
| *Castanopsis hystrix* | Control | 0.109±0.006^aB^ | 0.099±0.004^aB^ | 0.087±0.008^aB^ | 172.17±6.10^aB^ | 173.93±3.73^aB^ | 157.86±9.56^aB^ |
|  | MN | 0.085±0.007^bB^ | 0.092±0.007^aB^ | 0.089±0.006^aB^ | 156.06±5.54^aC^ | 168.18±5.39^aC^ | 165.54±5.32^aC^ |
|  | LN | 0.052±0.004^cB^ | 0.056±0.004^bB^ | 0.057±0.005^bB^ | 162.83±7.44^aB^ | 171.25±11.56^aB^ | 172.04±11.92^aB^ |
|  | *F* and *P* | *F*=23.593^***^, *P*<0.001 | *F*=19.902^***^, *P*<0.001 | *F*=7.669^**^, *P*=0.004 | *F*=1.592, *P*=0.231 | *F*=0.140, *P*=0.870 | *F*=0.578, *P*=0.571 |
| *Betula alnoides* | Control | 0.136±0.013^aA^ | 0.129±0.011^aA^ | 0.138±0.016^aA^ | 228.78±8.44^bA^ | 224.91±9.12^bA^ | 225.58±12.27^bA^ |
|  | MN | 0.125±0.007^aA^ | 0.128±0.007^aA^ | 0.129±0.007^aA^ | 227.34±11.03^bA^ | 233.60±9.61^bA^ | 233.77±9.86^bA^ |
|  | LN | 0.078±0.006^bA^ | 0.087±0.004^bA^ | 0.084±0.006^bA^ | 271.53±5.09^aA^ | 279.12±3.28^aA^ | 275.93±5.83^aA^ |
|  | *F* and *P* | *F*=11.233^**^, *P*=0.001 | *F*=9.113^**^, *P*=0.002 | *F*=7.344^**^, *P*=0.005 | *F*=8.643^**^, *P*=0.002 | *F*=13.648^***^, *P*<0.001 | *F*=7.770^**^, *P*=0.004 |

Statistical differences between each character of four species under different N treatments (Means±SE) results of a one-way ANOVA (n=7). Lower case letters indicate significant difference at 0.05 levels between N treatment and capital letter indicate significant difference at 0.05 levels between species under same N treatment. *F*-ratios with statistically significant values denoted by ^*^*P*<0.05, ^**^*P*<0.01, ^***^*P*<0.001 between N treatment. Control, high N; MN, medium N; LN, low N.

**Table S10** leaf C content per mass (*C*_mass_), C/N ratio, and chlorophyll contents (chlorophyll a, chlorophyll b and chlorophyll a+b) in four species seedling leaves leaf nitrogen content in four species seedling leaves under different soil nitrogen (N) treatments

| Tree species | N treatments | *C*_mass_ (mg g^-1^) | *C/N* (g g^–1^) | *Chl*a(mg g^-1^) | *Chl*b(mg g^-1^) | *Chl*a+b(mg g^-1^) |
| --- | --- | --- | --- | --- | --- | --- |
| *Dalbergia Odorifera* | Control | 449.49±8.86^aBC^ | 14.24±0.48^bC^ | 1.33±0.03^aA^ | 1.05±0.03^aA^ | 2.38±0.05^aA^ |
|  | MN | 467.87±10.75^aC^ | 16.02±1.00^abC^ | 1.15±0.01^bA^ | 0.82±0.01^bA^ | 1.97±0.03^bA^ |
|  | LN | 455.40±2.94^aC^ | 18.54±1.09^aC^ | 1.28±0.05^aA^ | 0.98±0.06^aA^ | 2.26±0.10^aA^ |
|  | *F* and *P* | *F*=1.302, *P*=0.296 | *F*=5.780^*^, *P*=0.012 | *F*=8.743^**^, *P*=0.002 | *F*=10.549^**^, *P*=0.001 | *F*=9.883^**^, *P*=0.001 |
| *Erythrophleum fordii* | Control | 516.65±13.98^aA^ | 18.70±1.05^aC^ | 0.75±0.11^aB^ | 0.56±0.09^bB^ | 1.31±0.20^bB^ |
|  | MN | 526.07±5.64^aA^ | 19.14±0.53^aC^ | 0.95±0.10^aAB^ | 0.80±0.10^abA^ | 2.22±0.19^aA^ |
|  | LN | 512.66±4.80^aA^ | 19.08±0.64^aC^ | 1.05±0.07^aB^ | 1.17±0.11^aA^ | 1.76±0.20^bB^ |
|  | *F* and *P* | *F*=0.568, *P*=0.577 | *F*=0.098, *P*=0.907 | *F*=2.681, *P*=0.096 | *F*=8.915^**^, *P*=0.002 | *F*=5.528^*^, *P*=0.013 |
| *Castanopsis hystrix* | Control | 479.65±4.66^aB^ | 47.02±1.09^bA^ | 0.35±0.04^aC^ | 0.33±0.04^aC^ | 0.68±0.08^aC^ |
|  | MN | 507.60±10.63^aAB^ | 53.15±1.73^abA^ | 0.39±0.05^aC^ | 0.35±0.05^aB^ | 0.74±0.10^aC^ |
|  | LN | 476.02±10.23^aBC^ | 55.28±1.45^aA^ | 0.34±0.05^aD^ | 0.30±0.04^aC^ | 0.63±0.10^aC^ |
|  | *F* and *P* | *F*=3.744^*^, *P*=0.044 | *F*=8.782^**^, *P*=0.002 | *F*=0.298, *P*=0.746 | *F*=0.355, *P*=0.706 | *F*=0.310, *P*=0.737 |
| *Betula alnoides* | Control | 493.63±5.40^aAB^ | 32.98±2.15^bB^ | 0.93±0.01^aB^ | 0.70±0.01^aB^ | 1.63±0.03^aB^ |
|  | MN | 487.60±4.72^aBC^ | 34.17±0.98^abBC^ | 0.77±0.02^cB^ | 0.57±0.01^bB^ | 1.34±0.03^bB^ |
|  | LN | 483.03±6.28^aB^ | 38.36±0.99^aB^ | 0.84±0.01^bC^ | 0.59±0.01^bB^ | 1.43±0.02^bB^ |
|  | *F* and *P* | *F*=0.933, *P*=0.411 | *F*=3.641^*^, *P*=0.047 | *F*=32.093^***^, *P*<0.001 | *F*=34.482^***^, *P*<0.001 | *F*=33.571^***^, *P*<0.001 |

Statistical differences between each character of four species under different N treatments (Means±SE) results of a one-way ANOVA (n=7). Lower case letters indicate significant difference at 0.05 levels between N treatment and capital letter indicate significant difference at 0.05 levels between species under same N treatment. *F*-ratios with statistically significant values denoted by ^*^*P*<0.05, ^**^*P*<0.01, ^***^*P*<0.001 between N treatment. Control, high N; MN, medium N; LN, low N.
